# Supplementary material for: Population sizes, HIV prevalence, and HIV prevention among men who paid for sex in sub-Saharan Africa (2000–2020): A meta-analysis of 87 population-based surveys
Source: PLoS Med. 2022 Jan 25;19(1):e1003861. doi: 10.1371/journal.pmed.1003861 (PMC8789156; doi:10.1371/journal.pmed.1003861)
Supplement: S1 Text — Table A: List of surveys considered and justifications for exclusion. Table B: Characteristics of population-based surveys conducted between 2000 and 2020 with available microdata included in analyses. Table C: Number of surveys, pooled estimates, confidence intervals, prediction intervals, and I2 values by region and overall for each outcome. Table D: Pooled estimates, confidence intervals, prediction intervals, and I2 for 2000–2009 and 2010–2020 for prevalence of paying for sex, condom use at last paid sex, and HIV prevalence and testing among men who have paid for sex. Table E: Results of univariate meta-regression for survey year. Table F: Pooled estimates, confidence intervals, prediction intervals, and I2 values for prevalence of paying for sex ever and in the past 12 months by urban/rural residence type. Table G: Pooled estimates, confidence intervals, prediction intervals, and I2 values for prevalence of paying for sex ever and in the past 12 months by age groups. Fig A: Flow charts of “HIV testing history” and “men who have ever paid for sex.” Fig B: Men ever paying for sex over time, by country. The proportion of sexually active men reporting ever paying for sex was calculated for 87 population-based surveys and plotted over time for countries with 3 or more surveys. Fig C: Bar graph of standardized mean lifetime number of sex partners for men who have paid for sex compared to men who have not, by survey. Fig D: Forest plot of proportion of men who paid for sex who reported condom use at last paid sex. Fig E: Condom use at last paid sex over time, by country. Fig F: Forest plot of standardized HIV prevalence for men who have paid for sex. Data from 52 population-based surveys was collected and meta-analysis conducted to determine HIV prevalence among men who reported having paid for sex. Prevalence is standardized by age and urban/ rural residence type. Proportions were pooled by region and overall. Fig G: Forest plot of standardized prevalence ratios for [file pmed.1003861.s001.docx]

**Supplementary material**

**HIV prevalence, population sizes, and HIV prevention among men who paid for sex in sub-Saharan Africa: a meta-analysis of 87 population-based surveys (2000-2020)**

Caroline Hodgins, James Stannah, Salome Kuchukhidze, Lycias Zembe, Jeffrey W Eaton, Marie-Claude Boily, Mathieu Maheu-Giroux

**SUPPLEMENTARY METHODS:**

**Data sources: Country-specific population-based surveys**

We reviewed five types of country-specific population-based surveys, including *Botswana AIDS Impact Surveys,* BAIS; *Eritrea Population and Health Survey,* EPHS; *Kenya AIDS Indicator Survey,* KAIS; *Nigeria HIV/AIDS Indicator and Impact Survey,* NAIIS; *South Africa National HIV Prevalence, Incidence, Behavior and Communication Survey;* SABSSM.

**Variables of interest and definitions: Paid sex**

Most surveys asked men if they had “*ever paid for sex*”. Two surveys asked men instead if they had “*ever given money, gifts, or favors in exchange for sex*” while another asked if they had ever had sex with a “*prostitute/ sex worker*” (Figure S2). Only nine surveys specified paying for sex with a woman, while the other surveys did not specify partner gender.

Two surveys (Cameroon 2004 DHS and Chad 2004 DHS) only asked men about paid sex ever if they reported sex with three or more women in the last 12 months; for these, we assumed that sexually active men with less than three recent partners had not paid for sex.

**Variables of interest and definitions: HIV testing**

HIV testing history was determined using questions asking if men had ever tested for HIV and the timing of their last test. If surveys asked men about receiving the results of their latest HIV test, only men who reported receiving their results were identified as having been tested. If men reported never having heard of HIV or AIDS, they were assumed to have never been tested.

**Variables of interest and definitions: Condom use**

Condom users during paid sex were defined as men who reported using a condom the most recent time they had paid for sex, which was asked only to men who paid for sex in the last 12 months (Figure S2).

**Table A. List of surveys considered and justifications for exclusion.**

| Country and year | Included or excluded | Justification for exclusion |
| --- | --- | --- |
| DEMOGRAPHIC AND HEALTH SURVEYS (DHS) | | |
| Angola 2015-16; Benin 2011-12, 2017-18; Burkina Faso 2010; Burundi 2010, 2016-17; Cameroon 2004, 2011, 2018; Chad 2004, 2014-15; Comoros 2012; Congo 2011-12; Côte d’Ivoire 2011-12; Democratic Republic of Congo 2013-14; Ethiopia 2000, 2005, 2011, 2016; Gabon 2000, 2012; Gambia 2013, 2019-20; Ghana 2003, 2014; Guinea 2005, 2012, 2018; Kenya 2003, 2014; Lesotho 2004, 2009, 2014; Liberia 2013, 2019-20; Madagascar 2003-04; Malawi 2000, 2004, 2010, 2015-16; Mali 2012-13, 2018; Mozambique 2003, 2011; Namibia 2000, 2013; Niger 2012; Nigeria 2003, 2013, 2018; Rwanda 2000, 2005, 2010, 2014-15; Senegal 2005, 2010-11, 2014, 2015, 2016, 2017, 2018, 2019; Sierra Leone 2013, 2019; South Africa 2016; Tanzania 2004-05, 2010; Togo 2013-14; Uganda 2000-01, 2011, 2016; Zambia 2001-02, 2013-14, 2018; Zimbabwe 2010-11, 2015 | Included | — |
| Benin 2001, 2006; Burkina Faso 2003; Congo 2005; Democratic Republic of Congo 2007; Eswatini 2006-07; Ethiopia 2019; Ghana 2008; Kenya 2008-09; Liberia 2007; Madagascar 2008-09; Mali 2001, 2006; Namibia 2006-07; Niger 2006; Nigeria 2008; Rwanda 2007-08; Sao Tome and Principe 2008-09; Senegal 2012-13; Sierra Leone 2008; Tanzania 2015-16; Uganda 2006; Zambia 2007; Zimbabwe 2005-06 | Excluded | No data on paid sex ever |
| Cape Verde 2005; Niger 2017 | Excluded | Not distributed |
| Equatorial Guinea 2011; South Africa 2003 | Excluded | Not in public domain |
| Eritrea 2002; Mauritania 2000-01 | Excluded | Restricted data |
| AIDS INDICATOR SURVEYS (AIS) | | |
| Mozambique 2015; Tanzania 2011-12 | Included | — |
| Congo 2009; Côte d’Ivoire 2005; Mozambique 2009; Tanzania 2003-04, 2007-08; Uganda 2011 | Excluded | No data on paid sex ever |
| Uganda 2004-05 | Excluded | Restricted data |
| POPULATION-BASED HIV IMPACT ASSESSMENT (PHIA) | | |
| Côte d’Ivoire 2017-18; Eswatini 2016-17; Lesotho 2016-17; Malawi 2015-16; Namibia 2017; Zambia 2016 | Included | — |
| Cameroon 2017-18; Ethiopia 2017-18; Rwanda 2018-19; Tanzania 2016-17; Uganda 2016-17; Zimbabwe 2015-16 | Excluded | No data on paid sex ever |
| BOTSWANA AIDS IMPACT SURVEYS (BAIS) | | |
| Botswana 2001, 2004, 2008, 2013 | Excluded | No data on paid sex ever |
| ERITREA POPULATION AND HEALTH SURVEY (EPHS) | | |
| Eritrea 2010 | Excluded | No data on paid sex ever |

**Table A continued...**

| KENYA AIDS INDICATOR SURVEY (KAIS) | | |
| --- | --- | --- |
| Kenya 2012 | Included | — |
| Kenya 2007 | Excluded | No data on paid sex ever |
| NIGERIA HIV/AIDS INDICATOR AND IMPACT SURVEY (NAIIS) | | |
| Nigeria 2018 | Excluded | No data on paid sex ever |
| SOUTH AFRICA NATIONAL HIV PREVALENCE, INCIDENCE, BEHAVIOR, AND COMMUNICATION SURVEY (SABSSM) | | |
| South Africa 2002, 2017 | Included | — |
| South Africa 2004-05, 2008-09, 2011-12 | Excluded | No data on paid sex ever |
| MULITPLE INDICATOR CLUSTER SURVEYS (MICS) | | |
| Angola 2001; Benin 2014; Botswana 2000; Burkina Faso 2006; Burundi 2000, 2005; Côte d’Ivoire 2000, 2006, 2016; Cameroon 2000, 2006, 2014; Central African Republic 2000, 2006, 2010, 2018-19; Chad 2000, 2010, 2019; Comoros 2000; Congo 2014-15; Democratic Republic of Congo 2001, 2010, 2017-18; Equatorial Guinea 2000; Eswatini 2000, 2010, 2014; Gambia 2000, 2005-06, 2010, 2018; Ghana 2006, 2007-08, 2010-11, 2011, 2017-18; Guinea 2016; Guinea-Bissau 2000, 2006, 2010, 2014, 2018-19; Kenya 2000, 2008, 2009, 2011, 2013-14, 2013-14, 2013-14; Lesotho 2000, 2018; Madagascar 2000, 2012, 2018; Malawi 2006, 2013-14; Mali 2009-10, 2015; Mauritania 2007, 2011, 2015; Mozambique 2008; Niger 2000; Nigeria 2007, 2011, 2016-17; Rwanda 2000; Sao Tome and Principe 2000, 2006, 2014, 2019; Senegal 2000, 2015-16; Sierra Leone 2000, 2005-06, 2010, 2017; South Sudan 2010; Togo 2000, 2006, 2010, 2017; Zimbabwe 2009, 2014, 2019 | Excluded | No data on paid sex ever |

**Table B. Characteristics of population-based surveys conducted between 2000 and 2020 with available microdata included in analyses.** Survey country, year, and type; sample size; and questions as written in published questionnaires are presented for 87 population-based surveys that asked men about paid sex ever. Surveys are organized by region, country, and ascending year. DHS: *Demographic and Health Survey*; AIS: *AIDS Indicator Survey*; PHIA: *Population-based HIV Impact Assessment*; KAIS: *Kenya AIDS Indicator Survey*; SABSSM: *South Africa National HIV Prevalence, Incidence, Behavior and Communication Survey*. Missing: question wording not available. NA: question not included in survey.

| Country | Year | Type | Sample Size of Sexually Active Men | Paid Sex in Last 12 Months | Paid Sex Ever | Condom Use During Paid Sex | Lifetime Sexual Partners | Heard of HIV/ AIDS | Ever Tested for HIV | Tested for HIV in Last 12 Months | Received Results of Last HIV Test |
| --- | --- | --- | --- | --- | --- | --- | --- | --- | --- | --- | --- |
| Central Africa | | | | | | | | | | | |
| Angola | 2015-16 | DHS | 5170 | Nos últimos 12 meses, o (NOME) pagou para manter relações sexuais com alguém? | Alguma vez pagou para manter relaçoes sexuais com alguém? | A última vez que pagou para ter relaçoes sexuais com alguém, usou, preservativo? | Em toda sua vida, com quantas, pessoas, teve relações sexuais? | Alguma vez ouviu falar de uma doença chamada VIH ou SIDA? | O senhor alguma vez fez teste de VIH? | Há quanto tempo fez seu último teste de VIH? | Recebeu os resultados do teste? |
| Cameroon | 2004 | DHS | 4275 | Au cours des 12 derniers mois, avez-vous payé quelqu'une pour avoir les rapports sexuels ? | missing | La dernière fois que vous avez payé quelqu'une pour avoir les rapports sexuels, est-ce qu'un condom a été utilisé ? | Au total, avec combien de personnes avez-vous eu des rapports sexuels dans votre vie ? | Avez-vous déjà entendu parler d'une maladie appelée SIDA ? | Je ne veux pas connaître les résultats, mais est-ce que vous avez déjà effectué un test pour voir si vous aviez le virus du SIDA ? | Quand avez-vous été testé pour la dernière fois ? | Je ne veux pas connaître les résultats du test, mais est-ce que vous avez obtenu les résultats ? |
| Cameroon | 2011 | DHS | 5277 | Au cours des 12 derniers mois, avez-vous payé une personne en échange de rapports sexuels ? | missing | La dernière fois que vous avez payé quelqu'une en échange de rapports sexuels, est-ce qu'un condom a été utilisé ? | En tout, avec combien de personnes différentes avez-vous eu des rapports sexuels dans toute votre vie ? | Avez-vous déjà entendu parler d'une maladie appelée sida? | Je ne veux pas connaître les résultats, mais avez-vous déjà effectué un test pour savoir si vous avez le sida ? | Quand avez-vous effectué le test du sida pour la dernière fois ? | Je ne veux pas connaître les résultats, mais avez-vous obtenu les résultats du test ? |
| Cameroon | 2018 | DHS | 5143 | Au cours des 12 derniers mois, avez-vous payé quelqu'une en échange de rapports sexuels ? | Est-ce qu'il vous est déjà arrivé de payer quelqu'un en échange de rapports sexuels ? | La dernière fois que vous avez payé quelqu'une en échange de rapports sexuels, un condom/préservatif a-t-il été utilisé ? | En tout, durant votre vie, avec combien de personnes différentes avez-vous eu des rapports sexuels ? | Avez-vous déjà entendu parler de VIH ou de sida ? | Avez-vous déjà effectué un test du VIH ? | Il y a combien de mois que vous avez effectué votre test du VIH le plus récent ? | Avez-vous eu les résultats du test? |
| Chad | 2004 | DHS | 1447 | Au cours des 12 derniers mois, avez-vous payé quelqu'une pour avoir les rapports sexuels ? | missing | La dernière fois que vous avez payé quelqu'une pour avoir les rapports sexuels, est-ce qu'un condom a été utilisé ? | Au total, avec combien de personnes avez-vous eu des rapports sexuels dans votre vie ? | Avez-vous déjà entendu parler d'une maladie appelée SIDA ? | Je ne veux pas connaître les résultats, mais est-ce que vous avez déjà effectué un test pour voir si vous aviez le virus du SIDA ? | Quand avez-vous été testé pour la dernière fois ? | Je ne veux pas connaître les résultats du test, mais est-ce que vous avez obtenu les résultats ? |
| Chad | 2014-15 | DHS | 3802 | Au cours des 12 derniers mois, avez-vous payé quelqu'un en échange de rapports sexuels ? | Avez-vous déjà payé quelqu'un en échange de rapports sexuels ? | La dernière fois que vous avez payé quelqu'un en échange de rapports sexuels, un condom a-t-il été utilisé? | En tout, durant votre vie, avec combien de personnes différentes avez-vous eu des rapports sexuels ? | Avez-vous délà entendu parler d'une maladie appelée sida? | Je ne veux pas connaître les résultats mais avez-vous déjà fait un test pour savoir si vous avez le virus du sida ? | Il y a combien de mois que vous avez effectué votre test du VIH le plus récent ? | je ne veux pas connaître les résultats mais avez-vous reçu les résultats du test ? |
| Congo | 2011-12 | DHS | 4591 | Au cours des 12 derniers mois, avez-vous payé quelqu'un en échange de rapports sexuels ? | Avez-vous déjà payé quelqu'un en échange de rapports sexuels ? | La dernière fois que vous avez payé quelqu'un en échange de rapports sexuels, un préservatif a-t-il été utilisé ? | En tout, durant votre vie, avec combien de personnes différentes avez-vous eu des rapports sexuels ? | Avez-vous délà entendu parler d'une maladie appelée sida? | Je ne veux pas connaître les résultats mais avez-vous déjà fait un test pour savoir si vous avez le virus du sida ? | Il y a combien de mois que vous avez effectué votre test du VIH le plus récent ? | je ne veux pas connaître les résultats mais avez-vous reçu les résultats du test ? |
| Congo Democratic Republic | 2013-14 | DHS | 7182 | Au cours des 12 derniers mois, avez-cous payé quelqu'un en échange de rapports sexuels ? | Avez-vous déjà payé quelqu'un en échange de rapports sexuels ? | La dernière fois que vous avez payé quelqu'un en échange de rapports sexuels, un condom a-t-il été utilisé? | En tout, durant votre vie, avec combien de personnes différentes avez-vous eu des rapports sexuels ? | Avez-vous déjà entendu parler d'une maladie appelée sida? | Je ne veux pas connaître les résultats mais avez-vous déjà fait un test pour savoir si vous avez le virus du sida ? | Il y a combien de mois que vous avez effectué votre test du VIH le plus récent ? | je ne veux pas connaître les résultats mais avez-vous reçu les résultats du test ? |

**Table B continued...**

| Gabon | 2000 | DHS | 1687 | Combien du temps s'est écoulé depuis la dernière fois que vous avez payé pour avoir des rapports sexuels avec une femme? | Vous est-il déjà arrivé de payer pour avoir des rapports sexuels avec une femme? | La dernière fois que vous avez payé pour avoir des rapports sexuels avec une femme, est-ce qu'une capote a été utilisée? | NA | Avez-vous déjà entendu parler d'une maladie appelée SIDA ? | Avez-vous déjà effectué un test pour savoir si vous aviez le virus du SIDA? | NA | NA |
| --- | --- | --- | --- | --- | --- | --- | --- | --- | --- | --- | --- |
| Gabon | 2012 | DHS | 5004 | Au cours des 12 derniers mois, avez-vous payé quelqu'un en échange de rapports sexuels ? | Avez-vous déjà payé quelqu'un en échange de rapports sexuels ? | La dernière fois que vous avez payé quelqu'un en échange de rapports sexuels, un préservatif a-t-il été utilisé ? | En tout, durant votre vie, avec combien de personnes différentes avez-vous eu des rapports sexuels ? | Avez-vous déjà entendu parler d'une maladie appelée sida? | Je ne veux pas connaître les résultats mais avez-vous déjà fait un test pour savoir si vous avez le virus du sida ? | Il y a combien de mois que vous avez effectué votre test du VIH le plus récent ? | je ne veux pas connaître les résultats mais avez-vous reçu les résultats du test ? |
| Western Africa | | | | | | | | | | | |
| Benin | 2011-12 | DHS | 4052 | Au cours des 12 derniers mois, avez-vous payé quelqu'un en échange de rapports sexuels ? | Avez-vous déjà payé quelqu'un en échange de rapports sexuels ? | La dernière fois que vous avez payé quelqu'un en échange de rapports sexuels, un condom a-t-il été utilisé? | En tout, durant votre vie, avec combien de personnes différentes avez-vous eu des rapports sexuels ? | Avez-vous délà entendu parler d'une maladie appelée sida? | Je ne veux pas connaître les résultats mais avez-vous déjà fait un test pour savoir si vous avez le virus du sida ? | Il y a combien de mois que vous avez effectué votre test du VIH le plus récent ? | je ne veux pas connaître les résultats mais avez-vous reçu les résultats du test ? |
| Benin | 2017-18 | DHS | 5843 | Au cours des 12 derniers mois, avez-vous payé quelqu'un en échange de rapports sexuels ? | Est-ce qu'il vous est déjà arrivé de payer quelqu'un en échange de rapports sexuels ? | La dernière fois que vous avez payé quelqu'un en échange de rapports sexuels, un condom a-t-il été utilisé? | En tout, durant votre vie, avec combien de personnes différentes avez-vous eu des rapports sexuels ? | Avez-vous déjà entendu parler de VIH ou de sida ? | Je ne veux pas connaître les résultats mais avez-vous déjà fait un test du VIH ? | Il y a combien de mois que vous avez effectué votre test du VIH le plus récent ? | Je ne veux pas connaître les résultats mais avez-vous eu les résultats du test ? |
| Burkina Faso | 2010 | DHS | 4996 | Au cours des 12 derniers mois, avez-vous payé quelqu'un en échange de rapports sexuels ? | Avez-vous déjà payé quelqu'un en échange de rapports sexuels ? | La dernière fois que vous avez payé quelqu'un en échange de rapports sexuels, un condom a-t-il été utilisé? | En tout, durant votre vie, avec combien de personnes différentes avez-vous eu des rapports sexuels ? | Avez-vous déjà entendu parler d'une maladie appelée sida? | Je ne veux pas connaître les résultats mais avez-vous déjà fait un test pour savoir si vous avez le virus du sida ? | Il y a combien de mois que vous avez effectué votre test du VIH le plus récent ? | je ne veux pas connaître les résultats mais avez-vous reçu les résultats du test ? |
| Côte d'Ivoire | 2011-12 | DHS | 4278 | Au cours des 12 derniers mois, avez-vous payé quelqu'un en échange de rapports sexuels ? | Avez-vous déjà payé quelqu'un en échange de rapports sexuels ? | La dernière fois que vous avez payé quelqu'un en échange de rapports sexuels, un condom a-t-il été utilisé? | En tout, durant votre vie, avec combien de personnes différentes avez-vous eu des rapports sexuels ? | Avez-vous déjà entendu parler d'une maladie appelée sida? | Je ne veux pas connaître les résultats mais avez-vous déjà fait un test pour savoir si vous avez le virus du sida ? | Il y a combien de mois que vous avez effectué votre test du VIH le plus récent ? | je ne veux pas connaître les résultats mais avez-vous reçu les résultats du test ? |
| Côte d’Ivoire | 2017-18 | PHIA | 7703 | In the last 12-months, have you paid money for sex? | Have you ever paid money for sex? | The last time you paid money for sex, was a condom used? | NA | NA | Have you ever tested for HIV? | What month and year was your last HIV test? | What was the result of that HIV test? |
| Gambia | 2013 | DHS | 2529 | In the last 12 months, did you pay anyone in exchange for having sexual intercourse? | Have you ever paid anyone in exchange for having sexual intercourse? | The last time you paid someone in exchange for having sexual intercourse, was a condom used? | In total, with how many different people have you had sexual intercourse in your lifetime? | Have you ever heard of an illness called AIDS? | I don't want to know the results, but have you ever been tested to see if you have the AIDS virus? | How many months ago was your most recent HIV test? | I don't want to know the results, but did you get the results of the test? |
| Gambia | 2019 | DHS | 3238 | In the last 12 months, did you pay anyone in exchange for having sexual intercourse? | Have you ever paid anyone in exchange for having sexual intercourse? | The last time you paid someone in exchange for having sexual intercourse, was a male condom or female condom used? | In total, with how many different people have you had sexual intercourse in your lifetime? | Have you ever heard of HIV or AIDS? | I don’t want to know the results, but have you ever been tested for HIV? | How many months ago was your most recent HIV test? | I don’t want to know the results, but did you get the results of the test? |
| Ghana | 2003 | DHS | 3695 | How long ago was the last time you paid for sex? | Have you ever paid for sex? | The last time that you paid for sex, was a condom used? | NA | Have you ever heard of an illness called AIDS? | I don't want to know the results, but have you ever been tested for the AIDS virus? | When was the last time you were tested? | I don't want to know the results, but did you get the results of the test? |
| Ghana | 2014 | DHS | 3327 | In the last 12 months, did you pay anyone in exchange for having sexual intercourse? | Have you ever paid anyone in exchange for having sexual intercourse? | The last time you paid someone in exchange for having sexual intercourse, was a condom used? | In total, with how many different people have you had sexual intercourse in your lifetime? | Have you ever heard of an illness called AIDS? | I don't want to know the results, but have you ever been tested to see if you have the AIDS virus? | How many months ago was your most recent HIV test? | I don't want to know the results, but did you get the results of the test? |
| Guinea | 2005 | DHS | 2606 | Au cours des 12 derniers mois, avez-vous payé quelqu'un en échange de rapports sexuels ? | missing | La dernière fois que vous avez payé quelqu'un en échange de rapports sexuels, un condom a-t-il été utilisé? | En tout, avec combien de personnes différentes avez-vous eu des rapports sexuels dans toute votre vie ? | Avez-vous déjà entendu parler dune maladie appelée SIDA ? | Je ne veux pas connaître les résultats, mais avez-vous déjà effectué un test pour savoir si vous aviez le sida ? | Quand avez-vous effectué le test du sida pour la dernière fois ? | Je ne veux pas connaître les résultats mais est-ce que vous avez obtenu les résultats du test ? |
| Guinea | 2012 | DHS | 3011 | Au cours des 12 derniers mois, avez-vous payé quelqu'un en échange de rapports sexuels ? | Avez-vous déjà payé quelqu'un en échange de rapports sexuels ? | La dernière fois que vous avez payé quelqu'un en échange de rapports sexuels, un condom a-t-il été utilisé? | En tout, durant votre vie, avec combien de personnes différentes avez-vous eu des rapports sexuels ? | Avez-vous délà entendu parler d'une maladie appelée sida ? | Je ne veux pas connaître les résultats mais avez-vous déjà fait un test pour savoir si vous avez le virus du sida ? | Il y a combien de mois que vous avez effectué votre test du VIH le plus récent ? | je ne veux pas connaître les résultats mais avez-vous reçu les résultats du test ? |

**Table B continued...**

| Guinea | 2018 | DHS | 3065 | Au cours des 12 derniers mois, avez-vous payé quelqu'un en échange de rapports sexuels ? | Est-ce qu'il vous est déjà arrivé de payer quelqu'un en échange de rapports sexuels ? | La dernière fois que vous avez payé quelqu'un en échange de rapports sexuels, un condom a-t-il été utilisé? | En tout, durant votre vie, avec combien de personnes différentes avez-vous eu des rapports sexuels ? | Avez-vous déjà entendu parler de VIH ou de sida ? | Je ne veux pas connaître les résultats mais avez-vous déjà fait un test du VIH ? | Il y a combien de mois que vous avez effectué votre test du VIH le plus récent ? | Je ne veux pas connaître les résultats mais avez-vous eu les résultats du test ? |
| --- | --- | --- | --- | --- | --- | --- | --- | --- | --- | --- | --- |
| Liberia | 2013 | DHS | 3564 | In the last 12 months, did you pay anyone in exchange for doing woman business? | Have you ever paid anyone in exchange for doing woman business? | The last time you paid someone in exchange for doing woman business, did you use a condom? | In your whole life, how many women have you done woman business with? | Have you ever heard of an illness called AIDS? | I don't want to know the results, but have you ever been tested to see if you have the AIDS virus? | How many months ago was your most recent HIV test? | I don't want to know the results, but did you get the results of the test? |
| Liberia | 2019-20 | DHS | 3558 | In the last 12 months, did you pay anyone in exchange for doing woman business? | Have you ever paid anyone in exchange for doing woman business? | The last time you paid someone for doing woman business, was a condom used? | In total, with how many different people have you done woman business in your lifetime? | Have you ever heard of HIV or AIDS? | I don’t want to know the results, but have you ever been tested for HIV? | How many months ago was your most recent HIV test? | I don’t want to know the results, but did you get the results of the test? |
| Mali | 2012-13 | DHS | 3421 | Au cours des 12 derniers mois, avez-vous payé quelqu'un en échange de rapports sexuels ? | Avez-vous déjà payé quelqu'un en échange de rapports sexuels ? | La dernière fois que vous avez payé quelqu'un en échange de rapports sexuels, un condom a-t-il été utilisé? | En tout, durant votre vie, avec combien de personnes différentes avez-vous eu des rapports sexuels ? | Avez-vous délà entendu parler d'une maladie appelée sida? | Je ne veux pas connaître les résultats mais avez-vous déjà fait un test pour savoir si vous avez le virus du sida ? | Il y a combien de mois que vous avez effectué votre test du VIH le plus récent ? | je ne veux pas connaître les résultats mais avez-vous reçu les résultats du test ? |
| Mali | 2018 | DHS | 3480 | Au cours des 12 derniers mois, avez-vous payé quelqu'un en échange de rapports sexuels ? | Est-ce qu'il vous est déjà arrivé de payer quelqu'un en échange de rapports sexuels ? | La dernière fois que vous avez payé quelqu'un en échange de rapports sexuels, un condom a-t-il été utilisé? | En tout, durant votre vie, avec combien de personnes différentes avez-vous eu des rapports sexuels ? | Avez-vous déjà entendu parler de VIH ou de sida ? | Je ne veux pas connaître les résultats mais avez-vous déjà fait un test du VIH ? | Il y a combien de mois que vous avez effectué votre test du VIH le plus récent ? | Je ne veux pas connaître les résultats mais avez-vous eu les résultats du test ? |
| Niger | 2012 | DHS | 2725 | Au cours des 12 derniers mois, avez-vous payé quelqu'un en échange de rapports sexuels ? | Avez-vous déjà payé quelqu'un en échange de rapports sexuels ? | La dernière fois que vous avez payé quelqu'un en échange de rapports sexuels, un condom a-t-il été utilisé? | En tout, durant votre vie, avec combien de personnes différentes avez-vous eu des rapports sexuels ? | Avez-vous déjà entendu parler d'une maladie appelée sida? | Je ne veux pas connaître les résultats mais avez-vous déjà fait un test pour savoir si vous avez le virus du sida ? | Depuis combien de mois avez-vous effectué votre test du VIH le plus récent ? | je ne veux pas connaître les résultats mais avez-vous reçu les résultats du test ? |
| Nigeria | 2003 | DHS | 1721 | How long ago was the last time you paid for sex? | Have you ever paid for sex? | The last time that you paid for sex, was a condom used on that occasion? | NA | Have you ever heard of an illness called AIDS? | I don’t want to know the results, but have you ever been tested to see if you have the AIDS virus? | When was the last time you were tested? | I don’t want to know the results, but did you get the results of the test? |
| Nigeria | 2013 | DHS | 12435 | In the last 12 months, did you pay anyone in exchange for having sexual intercourse? | Have you ever paid anyone in exchange for having sexual intercourse? | The last time you paid someone in exchange for having sexual intercourse, was a condom used? | In total, with how many different people have you had sexual intercourse in your lifetime? | Have you ever heard of an illness called AIDS? | I don't want to know the results, but have you ever been tested to see if you have the AIDS virus? | How many months ago was your most recent HIV test? | I don't want to know the results, but did you get the results of the test? |
| Nigeria | 2018 | DHS | 9385 | In the last 12 months, did you pay anyone in exchange for having sexual intercourse? | Have you ever paid anyone in exchange for having sexual intercourse? | The last time you paid someone in exchange for having sexual intercourse, was a condom used? | In total, with how many different people have you had sexual intercourse in your lifetime? | Have you ever heard of HIV or AIDS? | NA | NA | NA |
| Senegal | 2005 | DHS | 2301 | Au cours des 12 derniers mois, avez-vous payé quelqu'une en échange de rapports sexuels ? | missing | La dernière fois que vous avez payé quelqu'une en échange de rapports sexuels, un condom a-t-il été utilisé? | NA | Avez-vous déjà entendu parler dune maladie appelée SIDA ? | Je ne veux pas connaître les résultats, mais avez-vous déjà effectué un test pour savoir si vous aviez le sida ? | Quand avez-vous effectué le test du sida pour la dernière fois ? | Je ne veux pas connaître les résultats, mais est-ce que vous avez obtenu les résultats du test ? |
| Senegal | 2010-11 | DHS | 2712 | Au cours des 12 derniers mois, avez-vous payé quelqu'un en échange de rapports sexuels ? | Avez-vous déjà payé quelqu'un en échange de rapports sexuels ? | La dernière fois que vous avez payé quelqu'un en échange de rapports sexuels, un condom a-t-il été utilisé? | En tout, durant votre vie, avec combien de personnes différentes avez-vous eu des rapports sexuels ? | Avez-vous délà entendu parler d'une maladie appelée sida? | Avez-vous déjà fait un test pour savoir si vous avez le virus du sida ? | Il y a combien de mois que vous avez effectué votre test du VIH le plus récent ? | Avez-vous reçu les résultats du test ? |
| Senegal | 2014 | DHS | 1755 | Au cours des 12 derniers mois, avez-vous payé quelqu'un en échange de rapports sexuels ? | Avez-vous déjà payé quelqu'un en échange de rapports sexuels ? | La dernière fois que vous avez payé quelqu'un en échange de rapports sexuels, un condom a-t-il été utilisé? | En tout, durant votre vie, avec combien de personnes différentes avez-vous eu des rapports sexuels ? | Avez-vous déjà entendu parler d'une maladie appelée sida? | Avez-vous déjà fait un test pour savoir si vous avez le virus du sida ? | Il y a combien de mois que vous avez effectué votre test du VIH le plus récent ? | Avez-vous reçu les résultats du test ? |
| Senegal | 2015 | DHS | 2063 | Au cours des 12 derniers mois, avez-vous payé quelqu'un en échange de rapports sexuels ? | Avez-vous déjà payé quelqu'un en échange de rapports sexuels ? | La dernière fois que vous avez payé quelqu'un en échange de rapports sexuels, un condom a-t-il été utilisé ? | En tout, durant votre vie, avec combien de personnes différentes avez-vous eu des rapports sexuels ? | Avez-vous déjà entendu parler d'une maladie appelée sida? | Avez-vous déjà fait un test pour savoir si vous avez le virus du sida ? | Il y a combien de mois que vous avez effectué votre test du VIH le plus récent ? | Avez-vous reçu les résultats du test ? |
| Senegal | 2016 | DHS | 1799 | Au cours des 12 derniers mois, avez-vous payé quelqu'un en échange de rapports sexuels ? | Avez-vous déjà payé quelqu'un en échange de rapports sexuels ? | La dernière fois que vous avez payé quelqu'un en échange de rapports sexuels, un condom a-t-il été utilisé? | En tout, durant votre vie, avec combien de personnes différentes avez-vous eu des rapports sexuels ? | Avez-vous déjà entendu parler d'une maladie appelée sida? | Avez-vous déjà fait un test pour savoir si vous avez le virus du sida ? | Il y a combien de mois que vous avez effectué votre test du VIH le plus récent ? | Avez-vous reçu les résultats du test ? |

**Table B continued...**

| Senegal | 2017 | DHS | 4307 | Au cours des 12 derniers mois, avez-vous payé quelqu'un en échange de rapports sexuels ? | Est-ce qu'il vous est déjà arrivé de payer quelqu'un en échange de rapports sexuels ? | La dernière fois que vous avez payé quelqu'un en échange de rapports sexuels, un condom a-t-il été utilisé? | En tout, durant votre vie, avec combien de personnes différentes avez-vous eu des rapports sexuels ? | Avez-vous déjà entendu parler de VIH ou de sida ? | Je ne veux pas connaître les résultats mais avez-vous déjà fait un test du VIH ? | Il y a combien de mois que vous avez effectué votre test du VIH le plus récent ? | Je ne veux pas connaître les résultats mais avez-vous eu les résultats du test ? |
| --- | --- | --- | --- | --- | --- | --- | --- | --- | --- | --- | --- |
| Senegal | 2018 | DHS | 2149 | Au cours des 12 derniers mois, avez-vous payé quelqu'un en échange de rapports sexuels ? | Est-ce qu'il vous est déjà arrivé de payer quelqu'un en échange de rapports sexuels ? | La dernière fois que vous avez payé quelqu'un en échange de rapports sexuels, un condom a-t-il été utilisé? | En tout, durant votre vie, avec combien de personnes différentes avez-vous eu des rapports sexuels ? | NA | NA | NA | NA |
| Senegal | 2019 | DHS | 1935 | Au cours des 12 derniers mois, avez-vous payé quelqu'un en échange de rapports sexuels ? | Est-ce qu'il vous est déjà arrivé de payer quelqu'un en échange de rapports sexuels ? | La dernière fois que vous avez payé quelqu'un en échange de rapports sexuels, un condom a-t-il été utilisé? | En tout, durant votre vie, avec combien de personnes différentes avez-vous eu des rapports sexuels ? | NA | NA | NA | NA |
| Sierra Leone | 2013 | DHS | 5983 | In the last 12 months, did you pay anyone in exchange for having sexual intercourse? | Have you ever paid anyone in exchange for having sexual intercourse? | The last time you paid someone in exchange for having sexual intercourse, was a condom used? | In total, with how many different people have you had sexual intercourse in your lifetime? | Have you ever heard of an illness called AIDS? | I don't want to know the results, but have you ever been tested to see if you have the AIDS virus? | How many months ago was your most recent HIV test? | I don't want to know the results, but did you get the results of the test? |
| Sierra Leone | 2019 | DHS | 5851 | In the last 12 months, did you pay anyone in exchange for having sexual intercourse? | Have you ever paid anyone in exchange for having sexual intercourse? | The last time you paid someone in exchange for having sexual intercourse, was a condom used? | In total, with how many different people have you had sexual intercourse in your lifetime? | Have you ever heard of HIV or AIDS? | I don't want to know the results, but have you ever been tested for HIV? | How many months ago was your most recent HIV test? | I don't want to know the results, but did you get the results of the test? |
| Togo | 2013-14 | DHS | 3482 | Au cours des 12 derniers mois, avez-vous payé quelqu'un en échange de rapports sexuels ? | Avez-vous déjà payé quelqu'un en échange de rapports sexuels ? | La dernière fois que vous avez payé quelqu'un en échange de rapports sexuels, un condom a-t-il été utilisé? | En tout, durant votre vie, avec combien de personnes différentes avez-vous eu des rapports sexuels ? | Avez-vous délà entendu parler d'une maladie appelée sida? | Je ne veux pas connaître les résultats mais avez-vous déjà fait un test pour savoir si vous avez le virus du sida ? | Il y a combien de mois que vous avez effectué votre test du VIH le plus récent ? | je ne veux pas connaître les résultats mais avez-vous reçu les résultats du test ? |
| Eastern Africa | | | | | | | | | | | |
| Burundi | 2010 | DHS | 2865 | Au cours des 12 derniers mois, avez-vous payé quelqu'une en échange de rapports sexuels ? Muri aya mezi 12 aheze, vyoba vyarabashikiye mukariha umuntu amafaranga kugirango murangure amabanga mpuzabitsina ? | Avez-vous déjà payé quelqu'un en échange de rapports sexuels ? Vyoba bimaze kubashikira mukariha umuntu amafaranga kugirango murangure amabanga mpuzabitsina? | La dernière fois que vous avez payé quelqu'un en échange de rapports sexuels, un condom a-t-il été utilisé ? Aho muherukira kurangurana amabanga mpuzabitsina n'umuntu mwarishe amahera, mwoba mwarakoresheje agakingirizo ? | En tout, durant votre vie, avec combien de personnes différentes avez-vous eu des rapports sexuels ? Mu buzima bwawe umaze kurangurana amabanga mpuzabitsina n'abantu bangahe bose hamwe ? | Avez-vous délà entendu parler d'une maladie appelée sida ? Ubu naho nagomba duhindure tuvagane ivyerekeye ingwara ya SIDA. Mwoba mumaze kuyumva ? | Je ne veux pas connaître les résultats mais avez-vous déjà fait un test pour savoir si vous avez le virus du sida ? Sinipfuza kumenya inyishu, mugabo mwoba mumaze kwipimisha kugirango mumenye ko mufise umugera wa Sida ? | Il y a combien de mois que vous avez effectué votre test du VIH le plus récent ? Haheze amezi angahe mwipimishije umugera wa Sida ubuheruka ? | Je ne veux pas connaître les résultats mais avez-vous reçu les résultats du test ? Sinipfuza kumenya inyishu mugabo, mwoba mwaragiye gutora inyishu ? |
| Burundi | 2016-17 | DHS | 4926 | Muraya mezi 12 aheze, mwoba mwararanguranye amabanga mpuza bitsina n'umukenyezi mubanje kumuhonga? | Vyoba bimaze kubashikira mugahonga mushaka kurangurana amabanga mpuza bitsina? | None aho muherutse kurangura amabanga mpuza bitsina mubanje guhonga, mwoba mwarakoresheje agakingirizo? | Mu buzima bwawe umaze kurangurana amabanga mpuza bitsina n'abantu bangahe bose hamwe? | Ubu naho nagomba tuyage ibindi: Mwoba mumaze kwumva ibivurwa ku mugera wa Sida? | Sinshaka kumenya inyishu baguhaye, mugabo nagomba kukubaza mwoba mumaze kwipimisha kugirango mumenye ko mugendana umugera wa Sida ? | Haciye amezi angahe muherutse kwipimisha umugera wa Sida? | Sinipfuza kumenya inyishu babahaye mugabo mwoba mwararonse inyishu ? |
| Comoros | 2012 | DHS | 1578 | Au cours des 12 derniers mois, avez-vous payé quelqu'un en échange de rapports sexuels ? | Avez-vous déjà payé quelqu'un en échange de rapports sexuels ? | La dernière fois que vous avez payé quelqu'un en échange de rapports sexuels, un condom a-t-il été utilisé? | En tout, durant votre vie, avec combien de personnes différentes avez-vous eu des rapports sexuels ? | Avez-vous délà entendu parler d'une maladie appelée sida? | Je ne veux pas connaître les résultats mais avez-vous déjà fait un test pour savoir si vous avez le virus du sida ? | Il y a combien de mois que vous avez effectué votre test du VIH le plus récent ? | je ne veux pas connaître les résultats mais avez-vous reçu les résultats du test ? |
| Ethiopia | 2000 | DHS | 1787 | How long ago was the last time you paid for sex? | Have you ever paid for sex? | The last time you paid for sex, did you use a condom? | NA | Have you ever heard of an illness called AIDS? | Have you ever been tested for AIDS? | NA | NA |
| Ethiopia | 2005 | DHS | 3543 | In the last 12 months, did you pay anyone in exchange for sex? | missing | The last time you paid someone in exchange for sex, was a condom used? | NA | Have you ever heard of an illness called AIDS? | I don't want to know the results, but have you ever been tested to see if you have the AIDS virus? | When was the last time you were tested? | I don't want to know the results, but did you get the results of the test? |

**Table B continued...**

| Ethiopia | 2011 | DHS | 9545 | In the last 12 months, did you pay anyone in exchange for having sexual intercourse? | Have you ever paid anyone in exchange for having sexual intercourse? | The last time you paid someone in exchange for having sexual intercourse, was a male or female condom used? | In total, with how many different people have you had sexual intercourse in your lifetime? | Have you ever heard of an illness called AIDS? | I don't want to know the results, but have you ever been tested to see if you have the AIDS virus? | How many months ago was your most recent HIV test? | I don't want to know the results, but did you get the results of the test? |
| --- | --- | --- | --- | --- | --- | --- | --- | --- | --- | --- | --- |
| Ethiopia | 2016 | DHS | 8767 | In the last 13 monhts, did you pay anyone in exchange for having sexual intercourse? | Have you ever paid anyone in exchange for having sexual intercourse? | The last time you paid someone in exchange for having sexual intercourse, was a condom used? | In total, with how many different people have you had sexual intercourse in your lifetime? | Have you ever heard of HIV or AIDS? | I don't want to know the results, but have you ever been tested for HIV? | How many months ago was your most recent HIV test? | I don't want to know the results, but did you get the results of the test? |
| Kenya | 2003 | DHS | 3003 | How long ago was the last time you paid for sex? | Have you ever paid for sex? | The last time that you paid for sex, did you use a condom on that occasion? | NA | Have you ever heard of an illness called AIDS? | I do not want to know the results, but have you ever been tested to see if you have the AIDS virus? | When was the last time you were tested? | I do not want to know the results, but did you get the results of the test? |
| Kenya | 2012 | KAIS | 11454 | In the last 12-months, have you given money, gifts, or favors in exchange for sex? | Have you ever given money, gifts, or favors in exchange for sex? | The last time you gave money, gifts, or favors in exchange for sex, was a condom used? | In total, how many different people have you had sexual intercourse with in your lifetime? | Have you ever heard of an infection called HIV, the virus that causes AIDS? | Have you ever been tested for HIV? | When was your last HIV test? | What was the result of that HIV test? |
| Kenya | 2014 | DHS | 10728 | In the last 12 months, did you pay anyone in exchange for having sexual intercourse? | Have you ever paid anyone in exchange for having sexual intercourse? | The last time you paid someone in exchange for having sexual intercourse, was a condom used? | In total, with how many different people have you had sexual intercourse in your lifetime? | Have you ever heard of an illness called AIDS? | I don't want to know the results, but have you ever been tested to see if you have the AIDS virus? | How many months ago was your most recent HIV test? | I don't want to know the results, but did you get the results of the test? |
| Madagascar | 2003-04 | DHS | 1999 | Combien de temps s’est écoulé depuis la dernière fois que vous avez payé pour avoir des rapports sexuels avec une femme? | Vous est-il déjà arrivé de payer pour avoir des rapports sexuels avec une femme? | La dernière fois que vous avez payé pour avoir des rapports sexuels avec une femme, est-ce qu'un condom a été utilisé? | NA | Avez-vous déjà entendu parler dune maladie appelée SIDA? | Avez-vous déjà effectué un test pour savoir si vous aviez le virus du SIDA? | À quand remonte la dernière fois que vous avez effectué un test pour savoir si vous aviez le virus du SIDA? | Avez-vous obtenu le résultat du test pour savoir si vous aviez le virus du SIDA, mais je voudrais pas connaître le résultat? |
| Malawi | 2000 | DHS | 2485 | How long ago was the last time you paid for sex? | Have you ever paid for sex? | The last time that you paid for sex, was a condom used on that occasion? | NA | Have you ever heard of an illness called AIDS? | Have you ever been tested to see if you have the AIDS virus? | NA | NA |
| Malawi | 2004 | DHS | 2891 | How long ago was the last time you paid for sex? | Have you ever paid for sex? | The last time that you paid for sex, was a condom used on that occasion? | NA | Have you ever heard of an illness called AIDS? | I don't want to know the results, but have you ever been tested to see if you have the AIDS virus? | When was the last time you were tested? | I don't want to know the results, but did you get the results of the test? |
| Malawi | 2010 | DHS | 5795 | In the last 12 months, did you pay anyone in exchange for having sexual intercourse? | Have you ever paid for sex? | The last time you paid someone in exchange for having sexual intercourse, was a condom used? | In total, with how many different people have you had sexual intercourse in your lifetime? | Have you ever heard of an illness called AIDS? | Have you ever been tested to see if you have the AIDS virus? | When was the last time you were tested? | Did you get the results of the test? |
| Malawi | 2015-16 | PHIA | 6754 | In the last 12-months, have you paid money for sex? | Have you ever paid money for sex? | The last time you paid money for sex, was a condom used? | NA | NA | Have you ever tested for HIV? | What month and year was your last HIV test? | What was the result of that HIV test? |
| Malawi | 2015-16 | DHS | 6393 | In the last 12 months, did you pay anyone in exchange for having sexual intercourse? | Have you ever paid anyone in exchange for having sexual intercourse? | The last time you paid someone in exchange for having sexual intercourse, was a condom used? | In total, with how many different people have you had sexual intercourse in your lifetime? | Have you ever heard of HIV or AIDS? | I don't want to know the results, but have you ever been tested for HIV? | How many months ago was your most recent HIV test? | I don't want to know the results, but did you get the results of the test? |
| Mozambique | 2003 | DHS | 2462 | Quando foi a última vez que pagou por ter tido relações sexuais com uma mulher? | Alguma vez pagou por ter relações sexuais com uma mulher? | Da última vez que pagou para ter relações sexuais, usou preservativo? | NA | Alguma vez ouviu falar de HIV/SIDA? | Agora vamos falar sobre o teste de HIV/SIDA. Não estou interessado em saber o resultado. Já fez algum teste do SIDA? | Quando foi a última vez que fez teste do SIDA? | Não estou interessado em saber os resultados. Recebeu os resultados desse teste? |
| Mozambique | 2011 | DHS | 3436 | Nos últimos 12 meses pagou a alguém para manter relações sexuais? | Alguma vez pagou a alguém para manter relaçoes sexuais? | Da última vez que pagou para ter relações sexuais com alguém, usou preservativo? | No total, com quantas pessoas diferentes teve relações sexuais em toda a sua vida? | Alguma vez já ouviu falar de uma doença chamada SIDA? | Não estou interessada em saber o resultado, mas alguma vez foi testado para verificar se é portador do virus do SIDA? | Hà quantos meses fez o teste de HIV/SIDA mais recente? | Não estou interessado em saber o resultado, recebeu os resultados desse teste? |
| Mozambique | 2015 | AIS | 4589 | Nos últimos 12 meses, pagou a alguém para manter relações sexuais? | Alguma vez pagou a alguém para manter relaçoes sexuais? | A última vez que pagou para ter relações sexuais com alguém, usou preservativo? | Em toda sua vida, com quantas diferentes pessoas teve relações sexuais? | Alguma vez ouviu falar de uma doença chamada HIV/SIDA? | Alguma vez foi testado para verificar se é portador do virus do HIV/SIDA? | Há quantos meses foi seu teste mais recente? | Recebeu os resultados do teste? |

**Table B continued...**

| Rwanda | 2000 | DHS | 1535 | Il y a combien de temps que vous avez payé pour avoir des rapports sexuels? | Vous est-il déjà arrivé de payer pour avoir des rapports sexuels? | La dernière fois que vous avez payé pour avoir des rapports sexuels, est-ce qu'un condom a été utilisé? | NA | Avez-vous déjà entendu parler dune maladie appelée SIDA? | Avez-vous déjà effectué un test pour savoir si vous aviez le virus du SIDA? | NA | NA |
| --- | --- | --- | --- | --- | --- | --- | --- | --- | --- | --- | --- |
| Rwanda | 2005 | DHS | 3363 | In the last 12 months, did you pay anyone in exchange for sex? | Have you ever in your life paid someone in exchange for sex? | The last time you paid someone in exchange for sex, was a condom used? ** asked to all men who have paid for sex | In total, how many different people have you had sexual intercourse with in your lifetime? | Have you ever heard of an illness called AIDS? | I don’t want to know the results, but have you ever been tested to see if you have the AIDS virus? | When was the last time you were tested? | I don’t want to know the results, but did you get the results of the test? |
| Rwanda | 2010 | DHS | 3570 | In the last 12 months, did you pay anyone in exchange for having sexual intercourse? | Have you ever paid anyone in exchange for having sexual intercourse? | The last time you paid someone in exchange for having sexual intercourse, was a condom used? | In total, with how many different people have you had sexual intercourse in your lifetime? | Have you ever heard of an illness called AIDS? | I don't want to know the results, but have you ever been tested to see if you have the AIDS virus? | How many months ago was your most recent HIV test? | I don't want to know the results, but did you get the results of the test? |
| Rwanda | 2014-15 | DHS | 3674 | In the last 12 months, did you pay anyone in exchange for having sexual intercourse? | Have you ever paid anyone in exchange for having sexual intercourse? | The last time you paid someone in exchange for having sexual intercourse, was a condom used? | In total, with how many different people have you had sexual intercourse in your lifetime? | Have you ever heard of an illness called AIDS? | I don't want to know the results, but have you ever been tested to see if you have the AIDS virus? | How many months ago was your most recent HIV test? | I don't want to know the results, but did you get the results of the test? |
| Tanzania | 2004-05 | DHS | 1911 | In the last 12 months, did you pay anyone in exhange for sex? | Have you ever paid for sex? | The last time you paid someone in exchange for sex, was a condom used? | In total, with how many different people have you had sex in your lifetime? | Have you ever heard of an illness called AIDS? | I don't want to know the results, but have you ever been tested to see if you have the AIDS virus? | When was the last time you were tested? | I don't want to know the results, but did you get the results of the test? |
| Tanzania | 2010 | DHS | 1560 | In the last 12 months, did you pay anyone in exchange for having sexual intercourse? | Have you ever paid anyone in exchange for having sexual intercourse? | The last time you paid someone in exchange for having sexual intercourse, was a condom used? | In total, with how many different people have you had sexual intercourse in your lifetime? | Have you ever heard of an illness called AIDS? | I don't want to know the results, but have you ever been tested to see if you have the AIDS virus? | When was the last time you were tested? | I don't want to know the results, but did you get the results of the test? |
| Tanzania | 2011-12 | AIS | 6521 | In the last 12 months, did you pay anyone in exchange for having sexual intercourse? | Have you ever paid anyone in exchange for having sexual intercourse? | The last time you paid someone in exchange for sexual intercourse, was a condom used? | In total, with how many different people have you had sexual intercourse in your lifetime? | Have you ever heard of an illness called AIDS? | I don't want to know the results, but have you ever been tested to see if you have the AIDS virus? | How many months ago was your most recent HIV test? | I don't want to know the results, but did you get the results of the test? |
| Uganda | 2000-01 | DHS | 1452 | How long ago was the last time you paid for sex? | Have you ever paid for sex? | The last time that you paid for sex, was a condom used on that occasion? | NA | Have you ever heard of an illness called AIDS? | Have you ever been tested to see if you have the AIDS virus? | NA | Did you get the results? |
| Uganda | 2011 | DHS | 1902 | In the last 12 months, did you pay anyone in exchange for having sexual intercourse? | Have you ever paid anyone in exchange for having sexual intercourse? | The last time you paid someone in exchange for having sexual intercourse, was a condom used? | In total, with how many different people have you had sexual intercourse in your lifetime? | Have you ever heard of an illness called AIDS? | I don't want to know the results, but have you ever been tested to see if you have the AIDS virus? | How many months ago was your most recent HIV test? | I don't want to know the results, but did you get the results of the test? |
| Uganda | 2016 | DHS | 4464 | In the last 12 months, did you pay anyone in exchange for having sexual intercourse? | Have you ever paid anyone in exchange for having sexual intercourse? | The last time you paid someone in exchange for having sexual intercourse, was a condom used? | In total, with how many different people have you had sexual intercourse in your lifetime? | Have you ever heard of HIV or AIDS? | I don't want to know the results, but have you ever been tested for HIV? | How many months ago was your most recent HIV test? | I don't want to know the results, but did you get the results of the test? |
| Zambia | 2001-02 | DHS | 1686 | How long ago was the last time you paid for sex? | Have you ever paid for sex? | The last time that you paid for sex, was a condom used? | NA | Have you ever heard of a disease called AIDS? | Have you ever been tested to see if you have the AIDS virus? | NA | NA |
| Zambia | 2013-14 | DHS | 12318 | In the last 12 months, did you pay anyone in exchange for having sexual intercourse? | Have you ever paid anyone in exchange for having sexual intercourse? | The last time you paid someone in exchange for having sexual intercourse, was a female or male condom used? | In total, with how many different people have you had sexual intercourse in your lifetime? | Have you ever heard of an illness called AIDS? | I don't want to know the results, but have you ever been tested to see if you have the AIDS virus? | How many months ago was your most recent HIV test? | I don't want to know the results, but did you get the results of the test? |
| Zambia | 2016 | PHIA | 7539 | In the last 12 months, have you paid money for sex? | Have you ever paid money for sex? | The last time you paid money for sex, was a condom used? | In total, how many different people have you had sex with? Please give your best guess. | NA | Have you ever tested for HIV? | What month and year was your last HIV test? | What was the result of that HIV test? |
| Zambia | 2018 | DHS | 10004 | In the last 12 months, did you pay anyone in exchange for having sexual intercourse? | Have you ever paid anyone in exchange for having sexual intercourse? | The last time you paid someone in exchange for having sexual intercourse, was a condom used? | In total, with how many different people have you had sexual intercourse in your lifetime? | Have you ever heard of HIV or AIDS? | I don't want to know the results, but have you ever been tested for HIV? | How many months ago was your most recent HIV test? | I don't want to know the results, but did you get the results of the test? |
| Zimbabwe | 2010-11 | DHS | 5669 | In the last 12 months, did you pay anyone in exchange for having sexual intercourse? | Have you ever paid anyone in exchange for having sexual intercourse? | The last time you paid someone in exchange for having sexual intercourse, was a condom used? | In total, with how many people have you had sexual intercourse in your lifetime? | Have you ever heard of an illness called AIDS? | I don't want to know the results, but have you ever been tested to see if you have HIV? | How many months ago was your most recent HIV test? | I don't want to know the results, but did you get the results of the test? |

**Table B continued...**

| Zimbabwe | 2015 | DHS | 6554 | In the last 12 months, did you pay anyone in exchange for having sexual intercourse? | Have you ever paid anyone in exchange for having sexual intercourse? | The last time you paid someone in exchange for having sexual intercourse, was a condom used? | In total, with how many different people have you had sexual intercourse in your lifetime? | Have you ever heard of HIV or AIDS? | I don't want to know the results, but have you ever been tested for HIV? | How many months ago was your most recent HIV test? | I don't want to know the results, but did you get the results of the test? |
| --- | --- | --- | --- | --- | --- | --- | --- | --- | --- | --- | --- |
| Southern Africa | | | | | | | | | | |  |
| Eswatini | 2016-17 | PHIA | 3514 | In the last 12-months, have you paid money for sex? | Have you ever paid money for sex? | The last time you paid money for sex, was a condom used? | People often have sex with different people over their lifetime. In total, with how many different people have you had sex in your lifetime? Please give your best guess. | NA | Have you ever tested for HIV? | What month and year was your last HIV test? | What was the result of that test? |
| Lesotho | 2004 | DHS | 2680 | How long ago was the last time you paid for sex? | Have you ever paid for sex? | The last time that you paid for sex, was a male or female condom used on that occasion? ** 1 = male, 2 = female, 3 = no | NA | Have you ever heard of an illness called AIDS? | I don’t want to know the results, but have you ever been tested to see if you have the AIDS virus? | When was the last time you were tested? | I don’t want to know the results, but did you get the results of the test? |
| Lesotho | 2009 | DHS | 2295 | In the last 12 months, did you pay anyone in exchange for having sexual intercourse? | Have you ever paid anyone in exchange for having sexual intercourse? | The last time you paid someone in exchange for having sexual intercourse, was a male condom or female condom used? | In total, with how many different people have you had sexual intercourse in your lifetime? | Have you ever heard of an illness called AIDS? | I don't want to know the results, but have you ever been tested to see if you have the AIDS virus? | When was the last time you were tested? | I don't want to know the results, but did you get the results of the test? |
| Lesotho | 2014 | DHS | 2470 | In the last 12 months, did you pay anyone in exchange for having sexual intercourse? | Have you ever paid anyone in exchange for having sexual intercourse? | The last time you paid someone in exchange for having sexual intercourse, was a condom used? | In total, with how many different people have you had sexual intercourse in your lifetime? | Have you ever heard of an illness called AIDS? | I don't want to know the results, but have you ever been tested to see if you have HIV? | How many months ago was your most recent HIV test? | I don't want to know the results, but did you get the results of the test? |
| Lesotho | 2016-17 | PHIA | 4523 | In the last 12-months, have you paid money or given gifts for sex? | Have you ever paid money or given gifts for sex? | The last time you paid money or gave gifts for sex, was a condom used? | In total, with how many different people have you had sex in your lifetime? Please give your best guess. | NA | Have you ever tested for HIV? | What month and year waa your last HIV test? | What was the result of that HIV test? |
| Namibia | 2000 | DHS | 2235 | How long ago was the last time you paid for sex? | Have you ever paid for sex? | The last time you paid for sex, did you use a condom? | NA | Have you ever heard of an illness called AIDS? | I do not want to know the results, but have you ever been tested to see if you have the AIDS virus? | NA | I do not want you to tell me the results of the test, but have you been told the results? |
| Namibia | 2013 | DHS | 3577 | In the last 12 months, did you pay anyone in exchange for having sexual intercourse? | Have you ever paid anyone in exchange for having sexual intercourse? | The last time you paid someone in exchange for having sexual intercourse, was a condom used? | In total, with how many different people have you had sexual intercourse in your lifetime? | Have you ever heard of HIV/AIDS? | Have you ever been tested to see if you have HIV? | How many months ago was your most recent HIV test? | Did you get the results of the test? |
| Namibia | 2017 | PHIA | 6766 | In the last 12-months, have you paid money for sex? | Have you ever paid money for sex? | The last time you paid money for sex, was a condom used? | In total, with how many different people have you had sex in your lifetime? Please give your best guess. | Have you ever heard of HIV? | Have you ever tested for HIV? | What month and year was your last HIV test? | What was the result of that HIV test? |
| South Africa | 2002 | SABSSM | 2093 | NA | Have you ever had sex with a male or female prostitute/sex worker? | The last time you had sex with a prostitute/sex worker, did you use a condom? | NA | NA | Have you had an HIV test? | NA | Have you been told/informed of the result of this test? |
| South Africa | 2017 | SABSSM | 4906 | In the last 12-months, have you given money, gifts, or favours in exchange for sex? | Have you ever given money, gifts, or favours in exchange for sex? | The last time you gave money, gifts, or favours in exchange for sex, was a condomn used? (only for people who paid for sex in last 12 months) | How many people have you had sexual intercourse with in your lifetime? | NA | Have you ever had an HIV test? | How long ago did you have your most recent HIV test? | Have you been told/informed of the result of your most recent test? |
| South Africa | 2016 | DHS | 3018 | In the last 12 months, did you pay anyone in exchange for having sexual intercourse? | Have you ever paid anyone in exchange for having sexual intercourse? | The last time you paid someone in exchange for having sexual intercourse, was a condom used? | In total, with how many different people have you had sexual intercourse in your lifetime? | Have you ever heard of HIV or AIDS? | I don't want to know the results, but have you ever been tested for HIV? | How many months ago was your most recent HIV test? | I don't want to know the results, but did you get the results of the test? |

**Description of excluded surveys**

Condom use at last paid sex: 3 surveys had denominator < 10.

HIV prevalence and prevalence ratios: 2 surveys had insufficient data for men who paid for sex; 1 survey had concerns about accuracy of HIV serological assay; 1 survey could not link biomarker data to individuals; 1 survey collected HIV biomarker data but this was not yet available.

HIV testing ever: 3 surveys had insufficient data for men who paid for sex.

HIV testing ever among people living with HIV: 22 surveys had denominator <10; 13 surveys had insufficient data for men who paid for sex; 1 survey could not link biomarker data to individuals [29].

Antiretroviral use and viral load suppression: 1 survey had denominator <10.

All other surveys were included/ excluded based on inclusion of relevant data.

**Figure A. Flow chart of classification of:**

1. **HIV testing history**


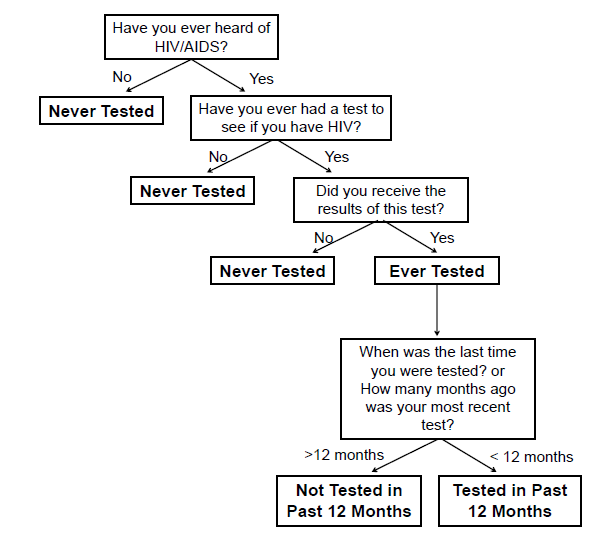


1.
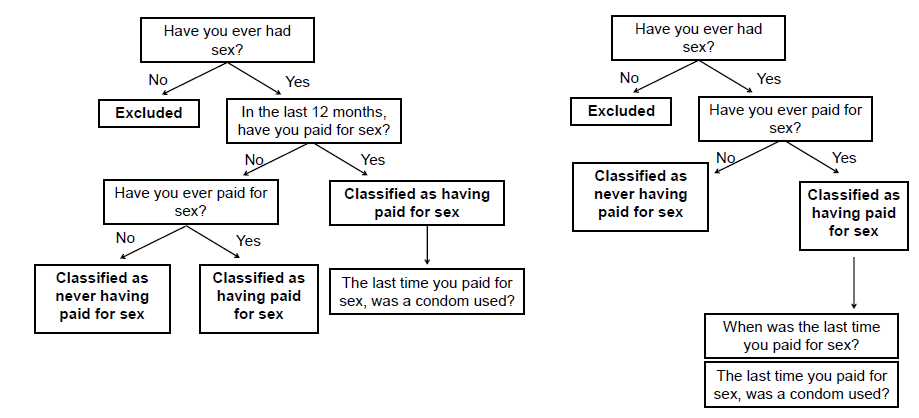
**Men who have ever paid for sex**

**Table C.** **Number of surveys, pooled estimates, confidence intervals, prediction intervals, and I^2^ by region and overall for each outcome.**

| Outcome | Region | Number of surveys | Pooled estimates | 95% Confidence interval | 95% Prediction interval | I^2^ (%) |
| --- | --- | --- | --- | --- | --- | --- |
| Proportion (%) of men who ever paid for sex | Central Africa | 10 | 11·9 | 4·4 to 28·7 | 0·8 to 69·4 | 99·8 |
|  | Western Africa | 31 | 5·6 | 4·3 to 7·5 | 1·3 to 21·5 | 99·2 |
|  | Eastern Africa | 35 | 11·3 | 7·6 to 16·4 | 1·7 to 48·7 | 99·8 |
|  | Southern Africa | 11 | 4·6 | 3·1 to 7·0 | 1·2 to 16·8 | 98·3 |
|  | Overall | 87 | 8·0 | 6·1 to 10·3 | 1·3 to 36·2 | 99·7 |
| Proportion (%) of men who paid for sex in the last 12 months | Central Africa | 7 | 6·1 | 3·9 to 9·4 | 1·8 to 18·7 | 98·5 |
|  | Western Africa | 29 | 1·8 | 1·1 to 2·8 | 0·2 to 12·2 | 98·9 |
|  | Eastern Africa | 33 | 3·6 | 2·4 to 5·5 | 0·3 to 36·6 | 99·7 |
|  | Southern Africa | 10 | 1·7 | 1·0 to 3·0 | 0·1 to 17·3 | 98·7 |
|  | Overall | 79 | 2·7 | 2·1 to 3·5 | 0·3 to 22·5 | 99·6 |
| Mean lifetime sexual partners of men who paid for sex ever | Central Africa | 9 | 19·6 | 15·5 to 24·8 | 9·4 to 40·9 | 100·0 |
|  | Western Africa | 27 | 10·8 | 9·4 to 12·4 | 4·8 to 24·5 | 100·0 |
|  | Eastern Africa | 24 | 10·2 | 9·1 to 11·3 | 6·1 to 16·9 | 100·0 |
|  | Southern Africa | 8 | 15·7 | 12·9 to 19·2 | 7·4 to 33·3 | 100·0 |
|  | Overall | 68 | 12·0 | 10·9 to 13·1 | 5·4 to 26·6 | 100·0 |
| Log transformed ratio of mean lifetime sexual partners among men who paid for sex and men who have not^1^ | Central Africa | 9 | 2·1 | 1·8 to 2·4 | 1·3 to 3·1 | 100·0 |
|  | Western Africa | 27 | 2·3 | 2·0 to 2·6 | 1·1 to 4·6 | 100·0 |
|  | Eastern Africa | 24 | 2·4 | 2·1 to 2·7 | 1·2 to 4·9 | 100·0 |
|  | Southern Africa | 8 | 2·1 | 1·8 to 2·6 | 1·2 to 3·8 | 100·0 |
|  | Overall | 68 | 2·3 | 2·1 to 2·4 | 1·2 to 4·2 | 100·0 |
| ^1^ standardized by age and urban/ rural residence type distribution among men who reported never paying for sex | | | | | | |

**Table C continued...**

| Outcome | Region | Number of surveys | Pooled estimates | 95% Confidence interval | 95% Prediction interval | I^2^ (%) |
| --- | --- | --- | --- | --- | --- | --- |
| Proportion (%) of condom use at last paid sex | Central Africa | 10 | 68·0 | 55·0 to 78·7 | 25·3 to 93·0 | 96·7 |
|  | Western Africa | 28 | 62·2 | 57·6 to 66·6 | 33·0 to 84·6 | 89·2 |
|  | Eastern Africa | 35 | 55·2 | 46·5 to 63·6 | 17·5 to 87·8 | 97·9 |
|  | Southern Africa | 11 | 76·6 | 65·8 to 84·7 | 32·6 to 95·7 | 87·8 |
|  | Overall | 84 | 62·2 | 57·4 to 66·7 | 24·8 to 89·1 | 96·6 |
| HIV prevalence (%) among men who paid for sex ever | Central Africa | 7 | 1·9 | 1·0 to 3·7 | 0·3 to 10·5 | 82·8 |
|  | Western Africa | 18 | 2·5 | 2·0 to 3·2 | 1·2 to 5·1 | 32·8 |
|  | Eastern Africa | 20 | 8·2 | 5·2 to 12·8 | 1·3 to 38·7 | 98·2 |
|  | Southern Africa | 7 | 18·8 | 4·3 to 54·6 | 0·2 to 96·7 | 98·3 |
|  | Overall | 52 | 5·1 | 3·4 to 7·5 | 0·4 to 39·8 | 98·0 |
| HIV prevalence ratio^1*^ | Central Africa | 7 | 1·21 | 0·90 to 1·63 | 0·66 to 2·22 | 29·0 |
|  | Western Africa | 18 | 1·67 | 1·10 to 2·53 | 0·41 to 6·79 | 63·1 |
|  | Eastern Africa | 20 | 1·62 | 1·35 to 1·94 | 0·85 to 3·06 | 85·1 |
|  | Southern Africa | 7 | 1·11 | 0·53 to 2·30 | 0·05 to 24·61 | 97·7 |
|  | Overall | 52 | 1·50 | 1·31 to 1·72 | 0·62 to 3·67 | 87·1 |
| HIV testing ever (%) among men who paid for sex ever | Central Africa | 10 | 25·7 | 10·7 to 50·0 | 0·4 to 96·5 | 99·8 |
|  | Western Africa | 28 | 25·2 | 21·6 to 29·1 | 6·9 to 60·6 | 96·4 |
|  | Eastern Africa | 35 | 42·2 | 27·2 to 58·7 | 2·6 to 95·3 | 99·7 |
|  | Southern Africa | 8 | 52·2 | 27·7 to 75·6 | 2·6 to 97·8 | 97·8 |
|  | Overall | 81 | 34·4 | 26·9 to 42·7 | 2·8 to 90·4 | 99·4 |
| ^1^ standardized by age and urban/ rural residence type distribution among men who reported never paying for sex  * prevalence ratios are for men who have ever paid for sex and men who have never paid for sex | | | | | | |

**Table C continued...**

| Outcome | Region | Number of surveys | Pooled estimates | 95% Confidence interval | 95% Prediction interval | I^2^ (%) |
| --- | --- | --- | --- | --- | --- | --- |
| HIV testing ever prevalence ratio^1*^ | Central Africa | 10 | 1·03 | 0·70 to 1·52 | 0·18 to 5·99 | 99·4 |
|  | Western Africa | 28 | 1·30 | 1·12 to 1·50 | 0·66 to 2·57 | 90·5 |
|  | Eastern Africa | 35 | 1·05 | 0·95 to 1·15 | 0·62 to 1·76 | 98·6 |
|  | Southern Africa | 8 | 1·08 | 0·95 to 1·23 | 0·87 to 1·34 | 52·2 |
|  | Overall | 81 | 1·14 | 1·06 to 1·24 | 0·62 to 2·10 | 98·1 |
| HIV testing in the last 12 months prevalence ratio^1*^ | Central Africa | 9 | 1·16 | 0·77 to 1·74 | 0·26 to 5·07 | 97·9 |
|  | Western Africa | 28 | 1·14 | 0·93 to 1·40 | 0·45 to 2·89 | 83·9 |
|  | Eastern Africa | 30 | 0·99 | 0·90 to 1·09 | 0·64 to 1·54 | 93·4 |
|  | Southern Africa | 9 | 1·12 | 1·08 to 1·17 | 1·08 to 1·17 | 0·0 |
|  | Overall | 76 | 1·09 | 1·00 to 1·18 | 0·57 to 2·07 | 94·7 |
| HIV testing ever among men living with HIV prevalence ratio^1*^ | Central Africa | 5 | 0·86 | 0·74 to 0·99 | 0·74 to 0·99 | 0·0 |
|  | Western Africa | 2 | 0·88 | 0·10 to 7·66 | 0·10 to 7·66 | 0·0 |
|  | Eastern Africa | 8 | 0·95 | 0·84 to 1·07 | 0·65 to 1·39 | 82·0 |
|  | Southern Africa | 3 | 0·76 | 0·19 to 3·01 | 0·06 to 9·41 | 98·5 |
|  | Overall | 18 | 0·96 | 0·88 to 1·05 | 0·68 to 1·37 | 83·3 |
| ^1^ standardized by age and urban/ rural residence type distribution among men who reported never paying for sex  * prevalence ratios are for men who have ever paid for sex and men who have never paid for sex | | | | | | |

**Table C continued...**

| Outcome | Region | Number of surveys | Pooled estimates | 95% Confidence interval | 95% Prediction interval | I^2^ (%) |
| --- | --- | --- | --- | --- | --- | --- |
| Antiretroviral (ARV) biomarkers among people living with HIV prevalence ratio^2*^ | Eastern Africa | 4 | 1·03 | 0·68 to 1·57 | 0·42 to 2·55 | 85·2 |
|  | Southern Africa | 4 | 0·98 | 0·74 to 1·31 | 0·61 to 1·60 | 49·0 |
|  | Overall | 8 | 1·01 | 0·86 to 1·18 | 0·66 to 1·54 | 70·7 |
| Viral load suppression (VLS) among people living with HIV prevalence ratio^2*^ | Eastern Africa | 4 | 0·97 | 0·77 to 1·22 | 0·64 to 1·48 | 50·2 |
|  | Southern Africa | 5 | 1·03 | 0·72 to 1·47 | 0·53 to 1·99 | 54·6 |
|  | Overall | 9 | 1·00 | 0·86 to 1·17 | 0·72 to 1·40 | 51·2 |
| ^2^ no surveys from Central Africa were available for this outcome; one survey from Western Africa had denominator <10 and was excluded  * prevalence ratios are for men who have ever paid for sex and men who have never paid for sex | | | | | | |

**Table D****. Pooled estimates, confidence intervals, prediction intervals, and I^2^ for 2000-2009 and 2010-2020 for prevalence of paying for sex, condom use at last paid sex, and HIV prevalence and testing among men who have paid for sex.**

| Outcome | Survey Year | Number of surveys | Pooled estimates (%) | 95% Confidence interval | 95% Prediction interval | I^2^ (%) |
| --- | --- | --- | --- | --- | --- | --- |
| Proportion (%) of men who ever paid for sex | 2010-2020 | 64 | 8·5 | 6·4 to 11·2 | 1·8 to 32·6 | 99·7 |
|  | 2000-2009 | 23 | 6·7 | 4·0 to 10·9 | 0·6 to 47·5 | 99·6 |
| Proportion (%) of condom use at last paid sex | 2010-2020 | 61 | 67·5 | 63·9 to 70·9 | 34·4 to 89·2 | 95·3 |
|  | 2000-2009 | 23 | 46·6 | 37·9 to 55·4 | 15·9 to 80·1 | 95·8 |
| HIV prevalence (%) among men who ever paid for sex | 2010-2020 | 42 | 4·5 | 3·0 to 6·8 | 0·4 to 37·1 | 98·3 |
|  | 2000-2009 | 10 | 8·7 | 3·8 to 18·8 | 0·6 to 59·3 | 94·3 |

**Table E.** **Results of univariate meta-regression for survey year.**

| Outcome | Slope of linear trend (on OR scale for proportions, log-transformed scale for ratios) | 95% Confidence interval | 2010 Prediction | 95% Confidence interval | 2020 Prediction | 95% Confidence interval |
| --- | --- | --- | --- | --- | --- | --- |
| Proportion (%) of men who ever paid for sex | 0·99 | 0·96 to 1·04 | 8·0% | 6·1 to 10·5% | 7·8% | 5·2 to 11·6% |
| Proportion (%) of condom use at last paid sex | 1·07 | 1·04 to 1·11 | 60·4% | 55·8 to 64·7% | 75·7% | 70·6 to 80·2% |
| HIV prevalence (%) among men who ever paid for sex | 0·98 | 0·95 to 1·01 | 5·5% | 3·7 to 8·2% | 3·6% | 1·6 to 8·1% |
| HIV prevalence ratio ^1^ | -0·02 | -0·06 to -0·01 | 1·59 | 1·33 to 1·90 | 1·25 | 1·01 to 1·54 |
| HIV testing ever (%) among men who ever paid for sex ^1^ | 1·15 | 1·10 to 1·20 | 31·9% | 25·5 to 39·1% | 64·9% | 52·0 to 75·9% |
| HIV testing ever prevalence ratio ^1^ | 0·00 | -0·01 to 0·01 | 1·14 | 1·04 to 1·25 | 1·15 | 1·00 to 1·33 |
| HIV testing in the last 12 months prevalence ratio ^1^ | -0·00 | -0·03 to 0·02 | 1·10 | 0·98 to 1·24 | 1·06 | 0·93 to 1·20 |
| ^1^standardized by age and urban/ rural residence type distribution among men who reported never paying for sex | | | | | | |

**Table F.** **Pooled estimates, confidence intervals, prediction intervals, and I^2^ for prevalence of paying for sex ever and in the past 12 months by urban/rural residence type.**

| Outcome | Number of surveys | Residence type | Pooled estimates (%) | 95% Confidence interval | 95% Prediction interval | I^2^ (%) |
| --- | --- | --- | --- | --- | --- | --- |
| Proportion (%) of men who ever paid for sex | 87 | Rural | 7·1 | 5·2 to 9·6 | 1·0 to 36·5 | 99·6 |
|  |  | Urban | 9·7 | 7·3 to 12·7 | 1·7 to 39·7 | 99·2 |
| Proportion (%) of men who paid for sex in past 12 months | 79 | Rural | 2·4 | 1·6 to 3·5 | 0·2 to 21·5 | 99·3 |
|  |  | Urban | 3·5 | 2·5 to 4·9 | 0·5 to 20·4 | 98·5 |

**Table G****. Pooled estimates, confidence intervals, prediction intervals, and I^2^ for prevalence of paying for sex ever and in the past 12 months by age groups.**

| Outcome | Number of surveys | Age group (years) | Pooled estimates (%) | 95% Confidence interval | 95% Prediction interval | I^2^ (%) |
| --- | --- | --- | --- | --- | --- | --- |
| Proportion (%) of men who ever paid for sex | 87 | 15-24 | 7·5 | 5·6 to 10·0 | 1·3 to 33·7 | 98·7 |
|  |  | 25-34 | 8·8 | 6·5 to 11·7 | 1·4 to 40·2 | 99·2 |
|  |  | 35-54 | 7·7 | 5·7 to 10·3 | 1·0 to 40·3 | 99·4 |
| Proportion (%) of men who paid for sex in past 12 months | 85 | 15-24 | 5·1 | 3·6 to 7·1 | 0·3 to 46·0 | 99·1 |
|  |  | 25-34 | 3·9 | 2·7 to 5·6 | 0·3 to 34·6 | 98·9 |
|  |  | 35-54 | 2·2 | 1·5 to 3·2 | 0·2 to 22·3 | 98·6 |

**Figure B****. Men ever paying for sex over time, by country.** Proportion of sexually active men reporting ever paying for sex was calculated for 87 population-based surveys and plotted over time for countries with three or more surveys.


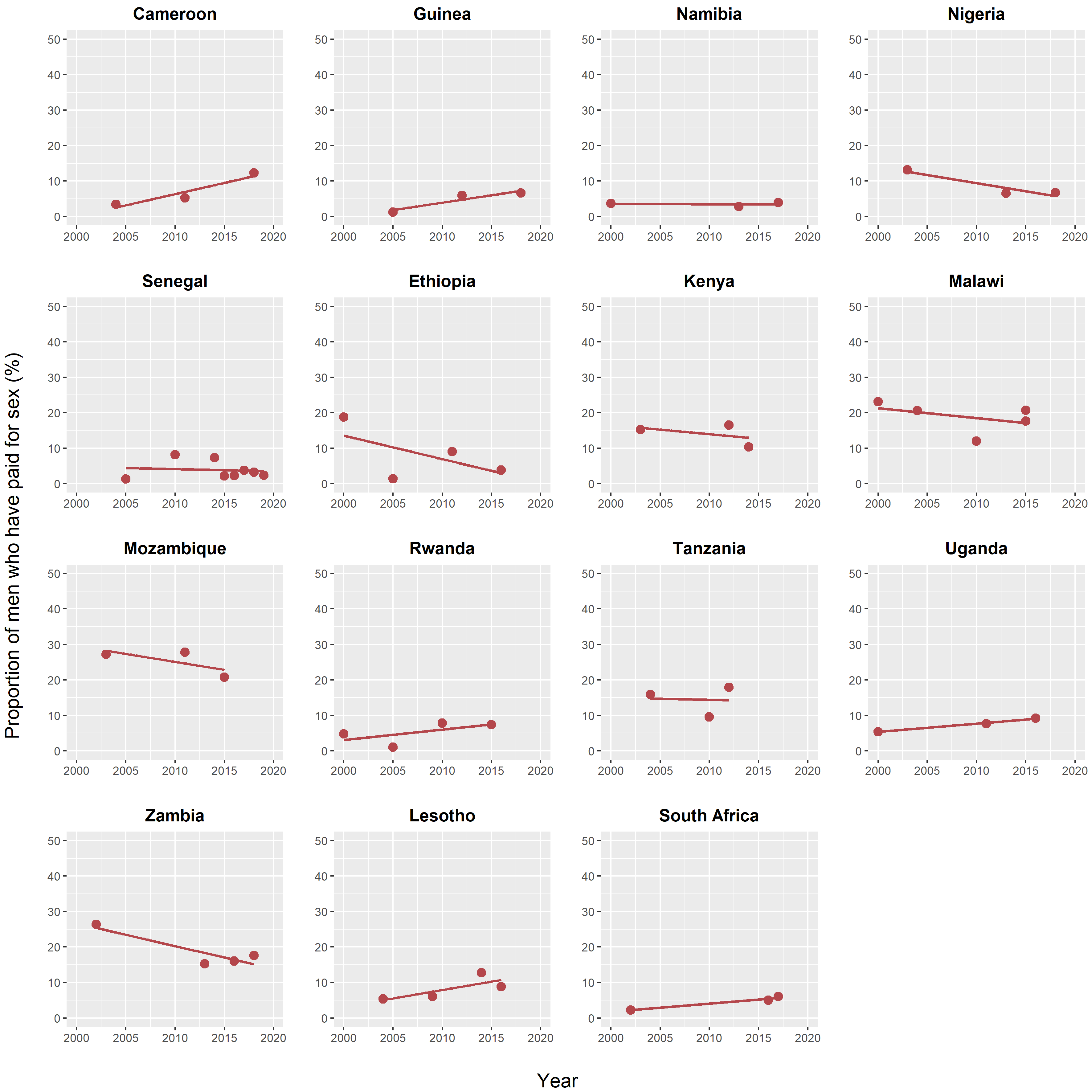


**Figure C.** **Bar graph of standardized mean lifetime sex partners for men who have paid for sex compared to men who have not by survey.** Data from 68 population-based surveys was collected and meta-analysis conducted to determine ratios of mean lifetime sex partners for men who reported having ever paid for sex compared to those who did not. Mean lifetime partners are standardized by age and urban/ rural residence type. Bar height represents mean lifetime sex partners. Whiskers represent 95% confidence intervals. Blue bars represent mean partners for men who paid for sex, while yellow bars represent mean partners for men who never paid for sex.

**
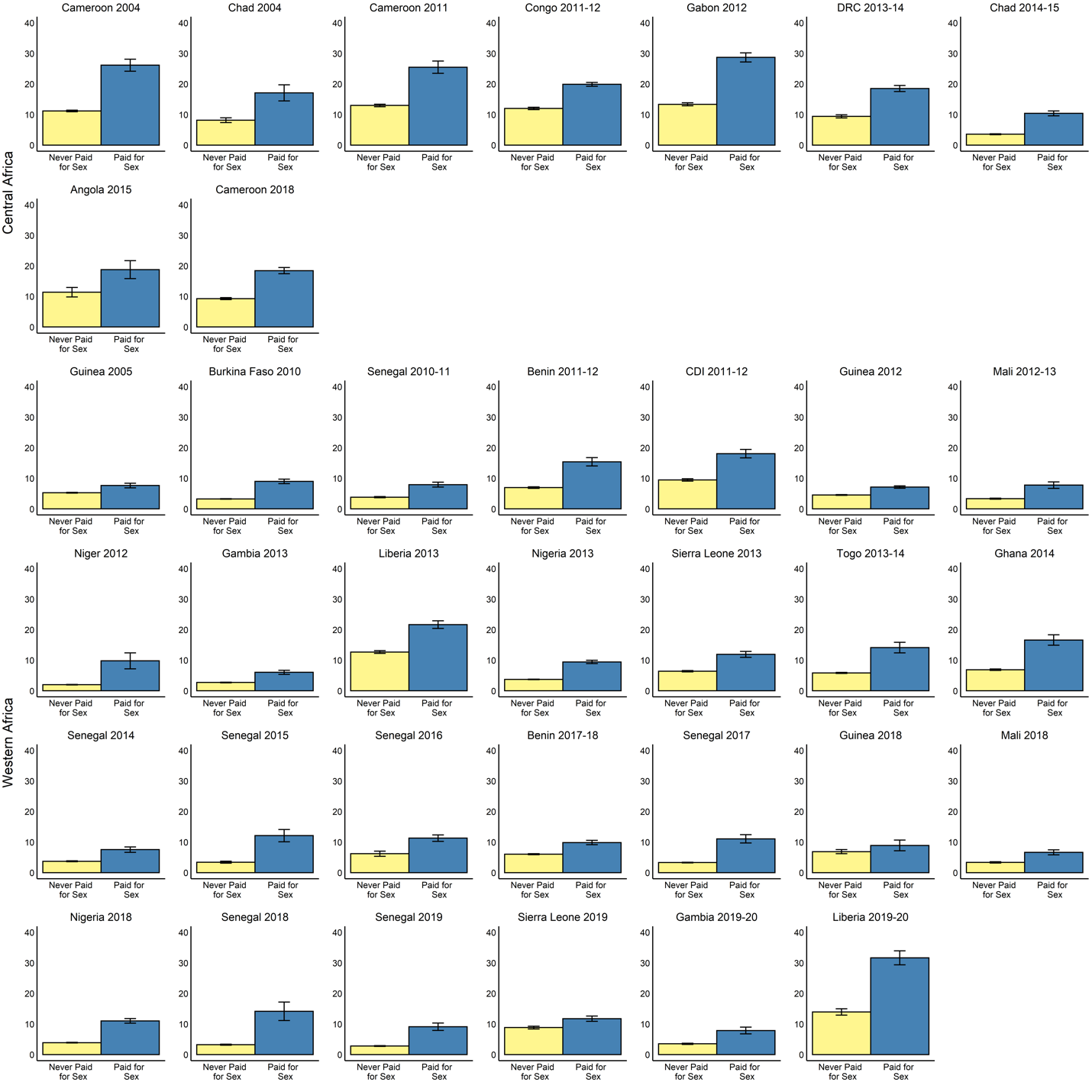
**

**
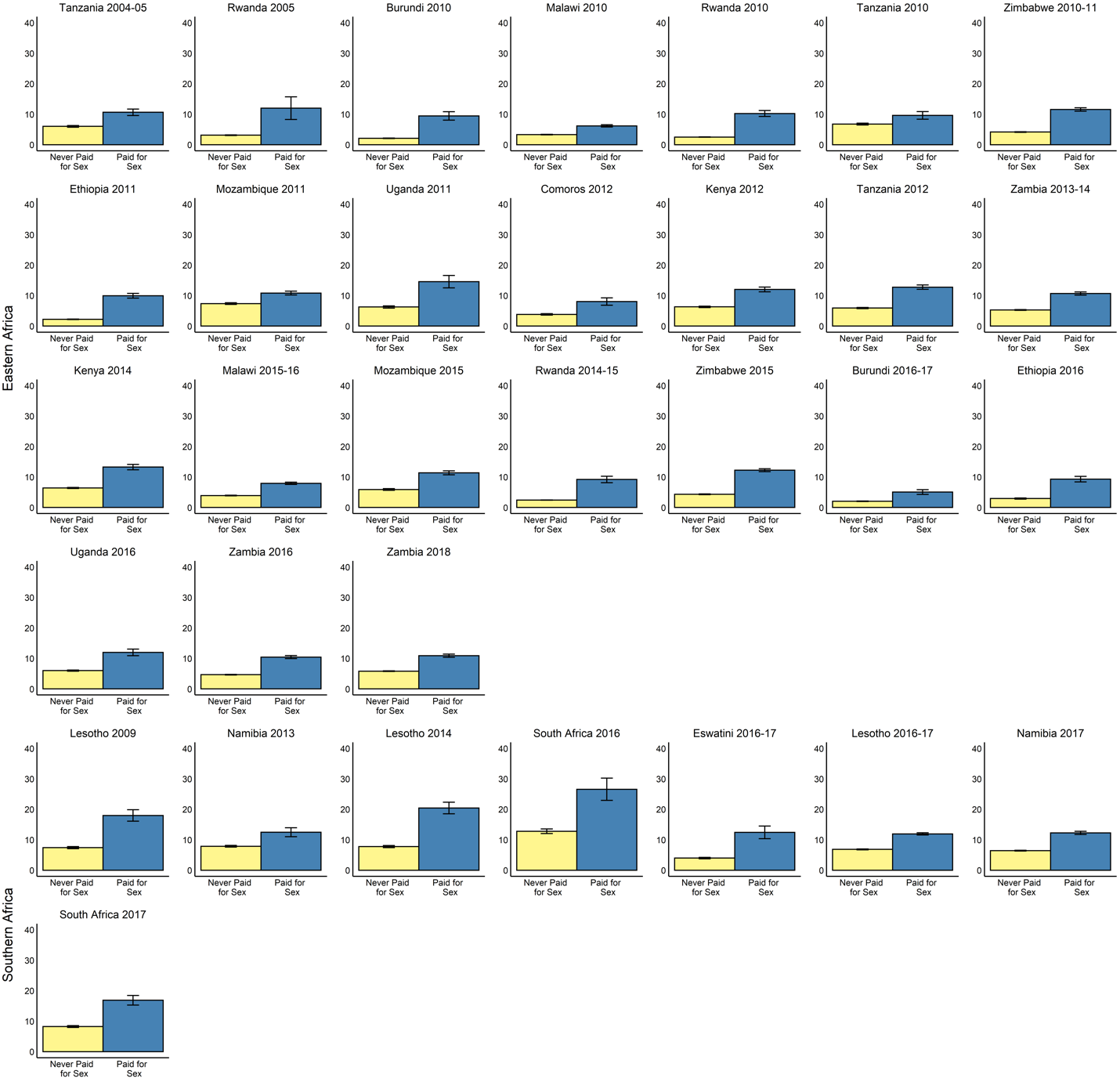
**

**Figure D.** **Forest plot of proportion of men who paid for sex who reported condom use at last paid sex.** Data from 84 population-based surveys was collected and meta-analysis conducted to determine proportion of men who paid for sex in the last 12 months and used a condom the last time they paid for sex. Proportions were pooled by region and overall.


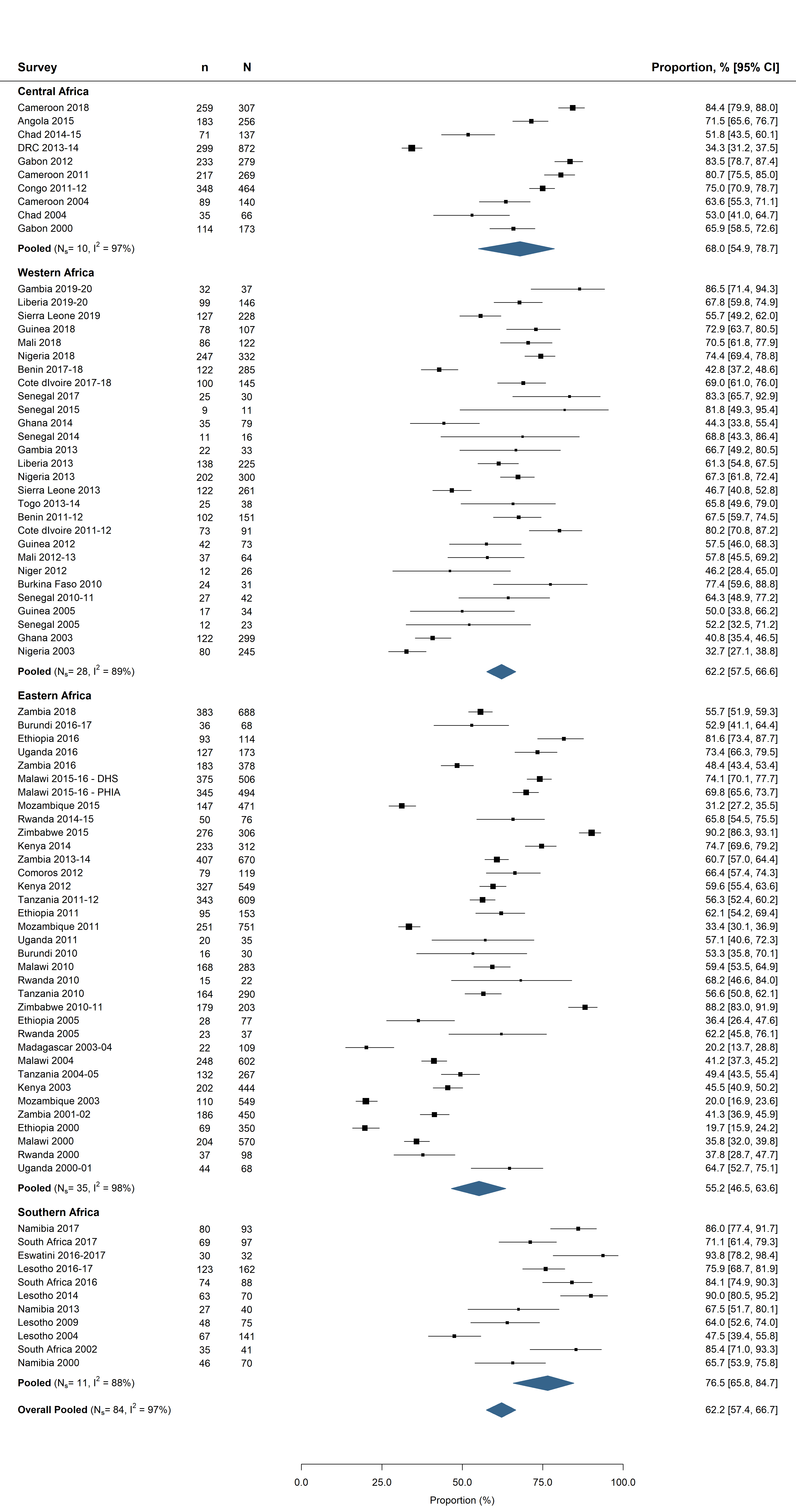


**Figure E.** **Condom use at last paid sex over time, by country.** For men who reported paying for sex in the last 12 months, the proportion of men reporting condom use at last paid sex was calculated for 84 population-based surveys. Proportions were plotted over time for countries with three or more surveys.

**
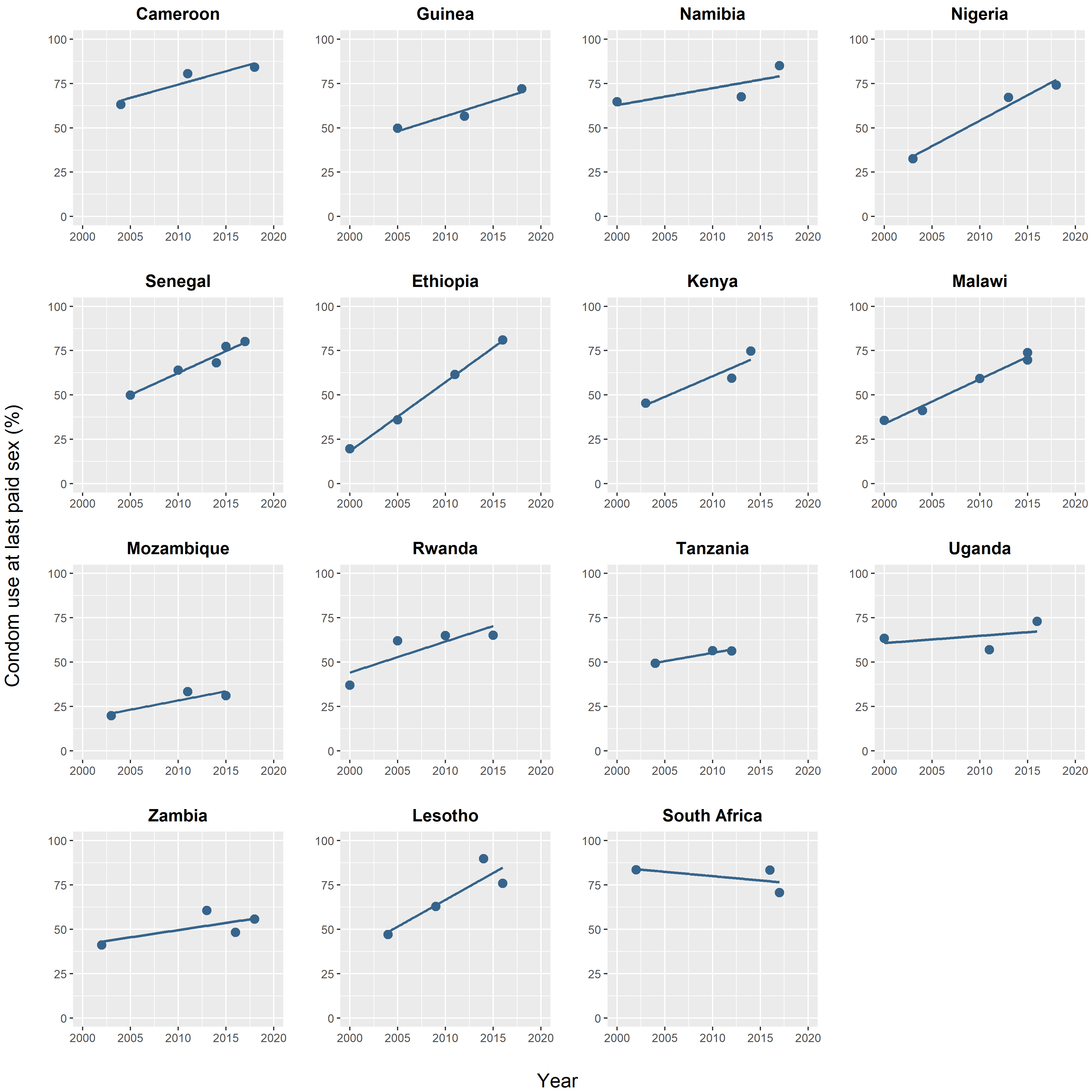
**

**Figure F.** **Forest plot of standardized HIV prevalence for men who have paid for sex.** Data from 52 population-based surveys was collected and meta-analysis conducted to determine HIV prevalence among men who reported having paid for sex. Prevalence is standardized by age and urban/ rural residence type. Proportions were pooled by region and overall.


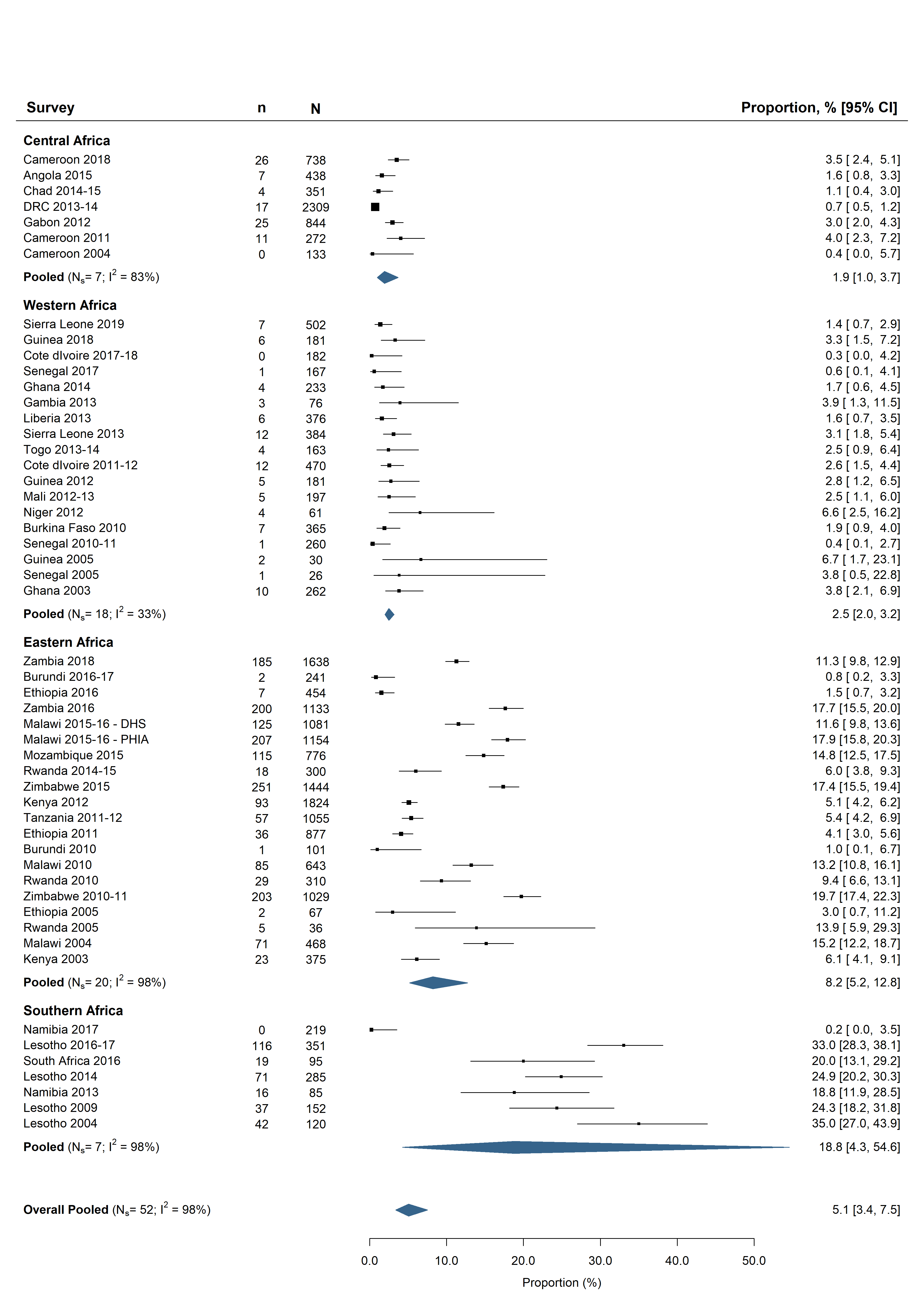


**Figure G.** **Forest plot of standardized prevalence ratios for HIV testing ever among men who have paid for sex compared to men who have not.** Data from 81 population-based surveys was collected and meta-analysis conducted to determine prevalence ratios (PR) of HIV testing ever for men who paid for sex compared to men who have not. PR are standardized by age and urban/ rural residence type. PR were pooled by region and overall.


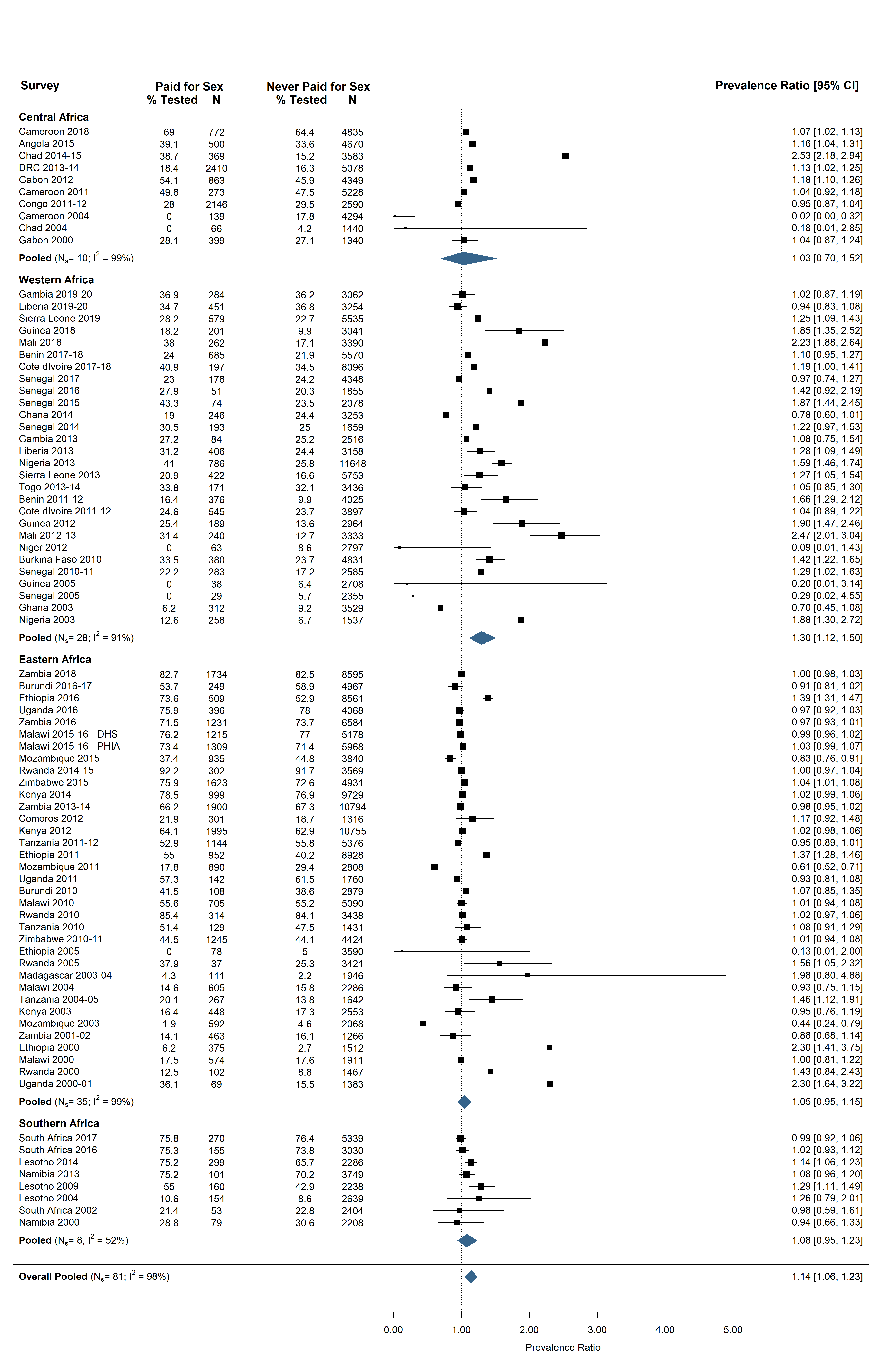


**Figure H.** **Forest plot of standardized prevalence ratios for HIV testing in the last 12 months among men who have paid for sex compared to men who have not.** Data from 76 population-based surveys was collected and meta-analysis conducted to determine prevalence ratios (PR) of HIV testing in the last 12 months for men who paid for sex compared to men who have not. PR are standardized by age and urban/ rural residence type. PR were pooled by region and overall.


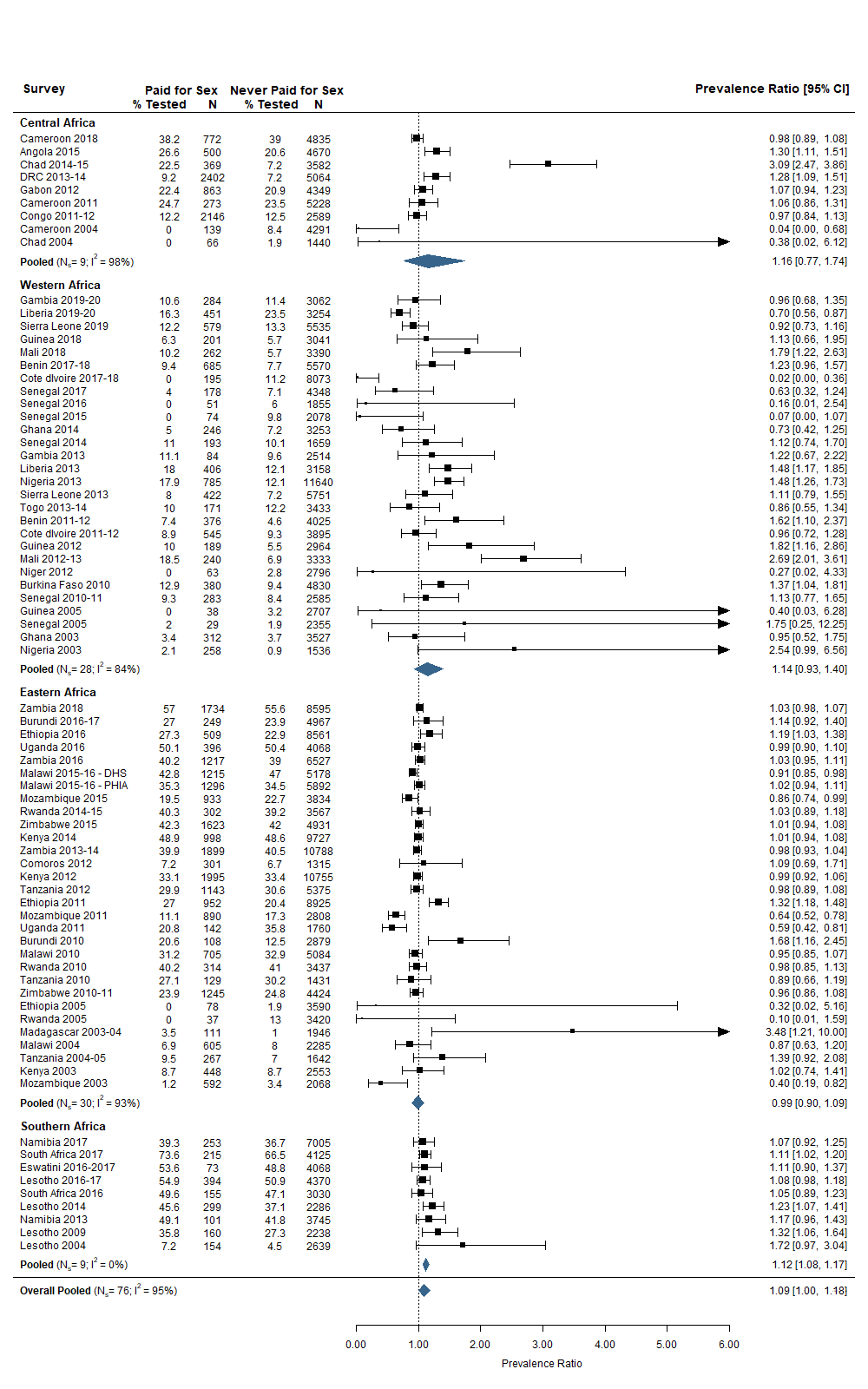


**Figure I.** **Forest plot of standardized prevalence ratios for HIV testing ever among people living with HIV (PLHIV) for men who have paid for sex compared to men who have not.** Data from 18 population-based surveys was collected and meta-analysis conducted to determine prevalence ratios (PR) of HIV testing ever for PLHIV who have paid for sex and PLHIV who have not. PR are standardized by age and urban/ rural residence type. PR were pooled by region and overall.


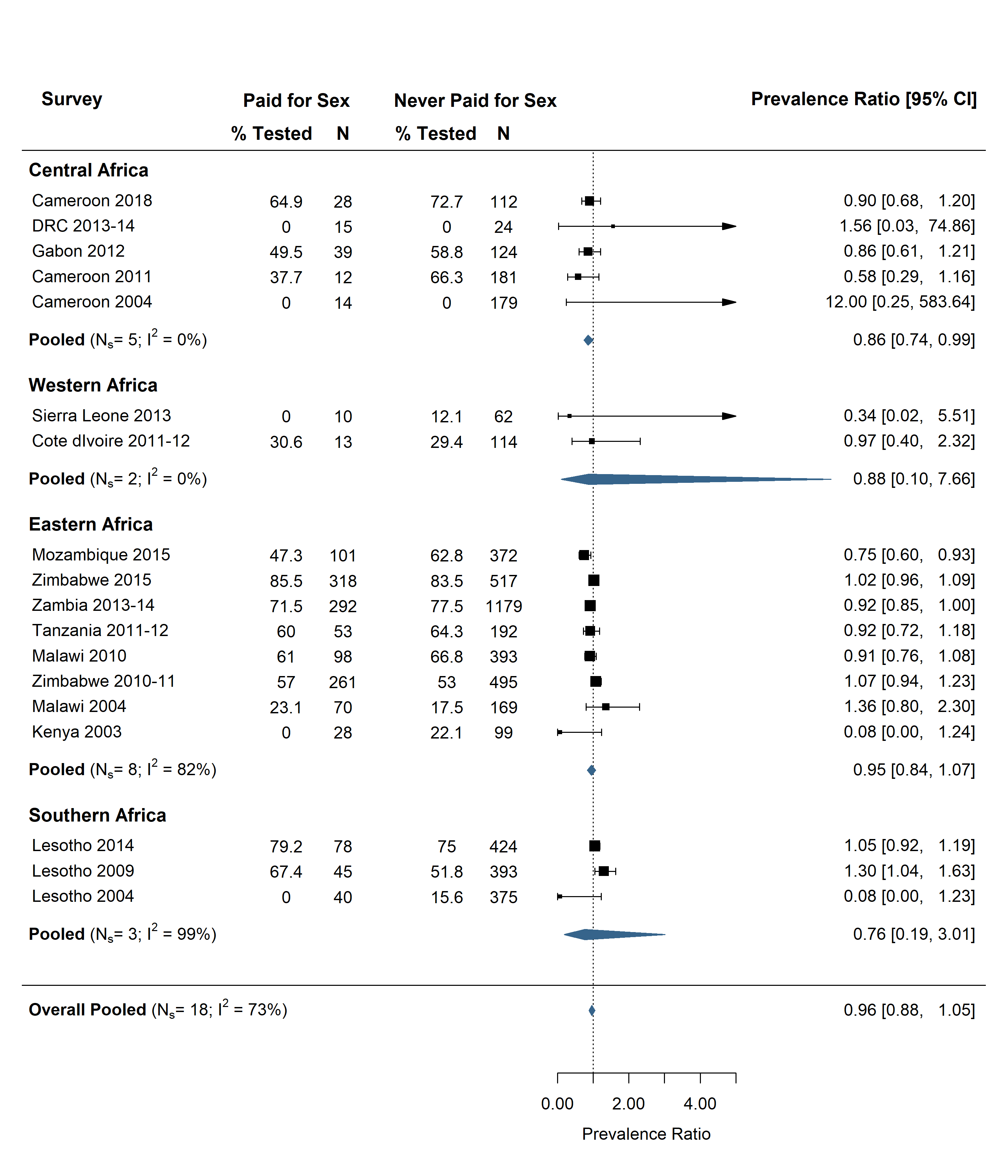


**Figure J.** **Forest plot of prevalence ratios of antiretroviral (ARV) use among people living with HIV for men who have paid for sex compared to men who have not.** Data from 8 population-based surveys was collected and meta-analysis conducted to determine prevalence ratios (PR) of ARV use for men who have paid for sex living with HIV compared with men living with HIV who have never paid for sex. PR were pooled by region and overall.


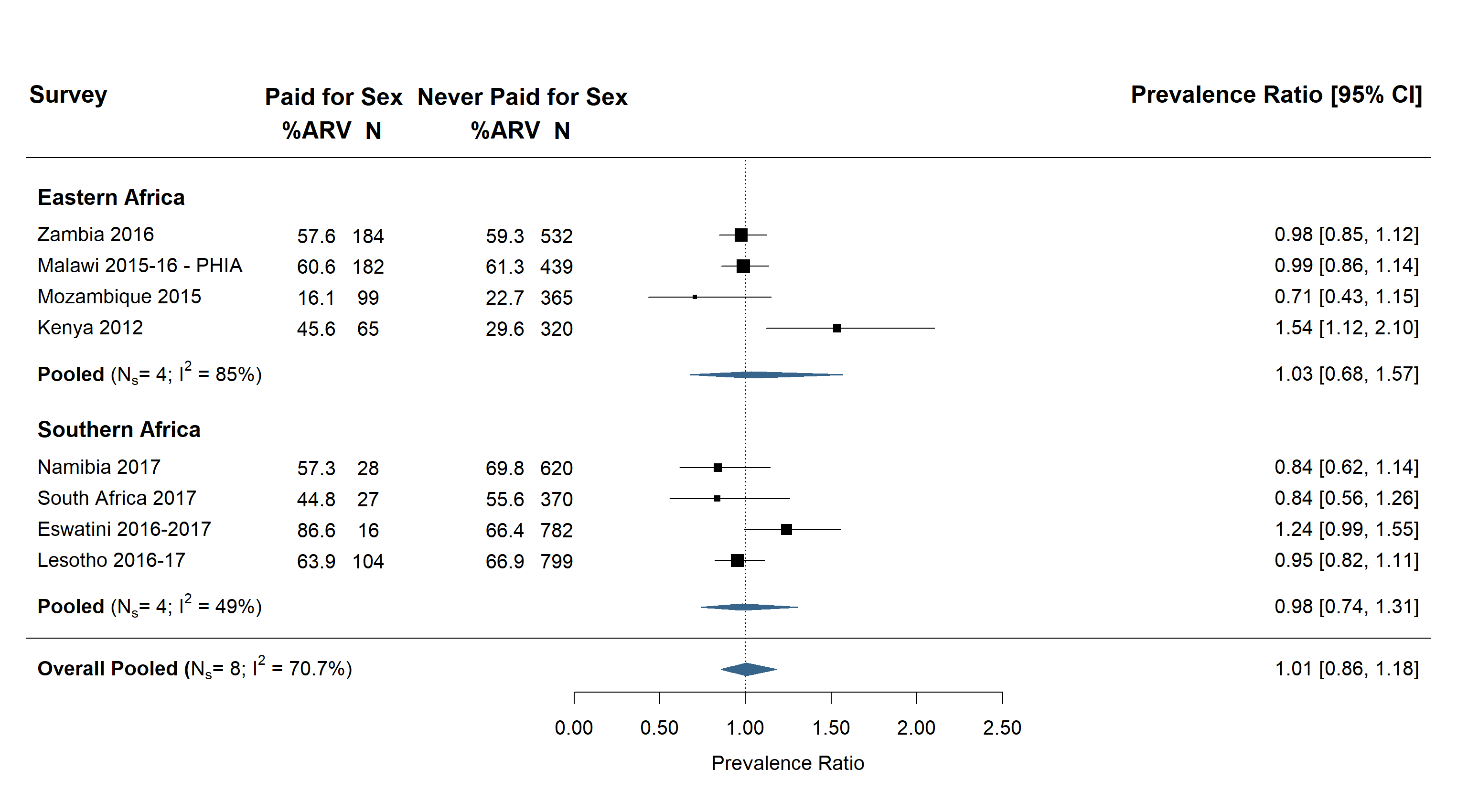


**Figure K.** **Forest plot of prevalence ratios of viral load suppression (VLS) among people living with HIV for men who have paid for sex compared to men who have not.** Data from 9 population-based surveys was collected and meta-analysis conducted to determine prevalence ratios (PR) of VLS for men who have paid for sex compared to men who have not living with HIV. PR were pooled by region and overall.

**
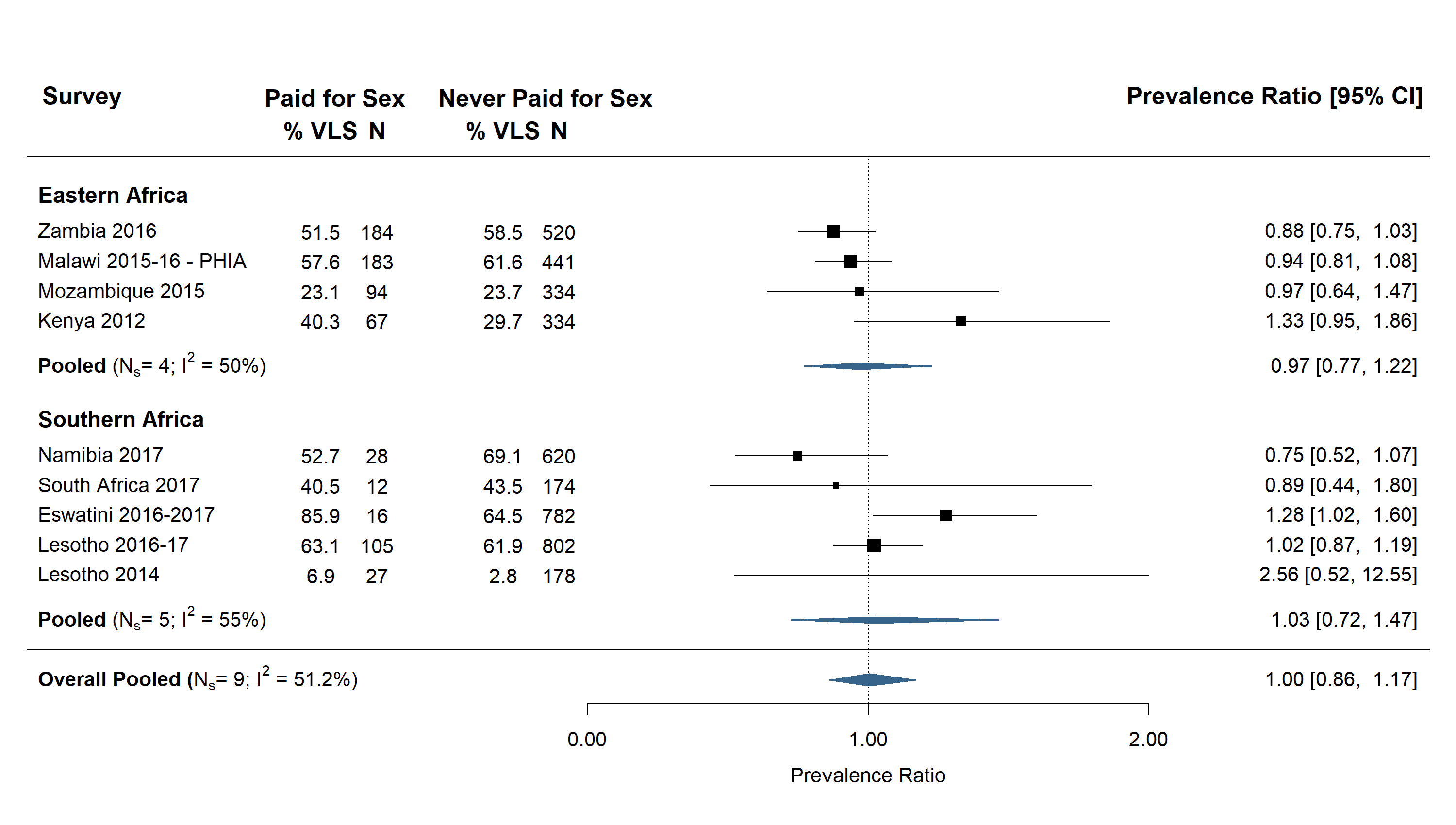
**

**SUPPLEMENTARY REFERENCES**

1. Instituto Nacional de Estatistica (INE) [Angola], Ministério da Saúde (MINSA) [Angola] Ministério do Planeamento e do Desenvolvimento Territorial (MINPLAN) [Angola] e ICF. 2017. *Inquérito de Indicadores Múltiplos e de Saúde em Angola 2015-2016* [Dataset]*.* AOMR71.DTA. Luanda, Angola e Rockville, Maryland, EUA: INE, MINSA, MINPLAN e ICF [Producers]. ICF [Distributor], 2017.
2. Institut National de la Statistique (INS) [Cameroon] et ORC Macro. 2004. *Enquête Démographique et de Santé du Cameroun 2004* [Dataset]. CMMR44.DTA. Calverton, Maryland, USA: INS et ORC Macro [Producers]. ICF [Distributor], 2004.
3. Institut National de la Statistique (INS) [Cameroon] et ICF International. 2012. *Enquête Démographique et de Santé et à Indicateurs Multiples du Cameroun 2011* [Dataset]. CMMR61.DTA. Calverton, Maryland, USA: INS et ICF International [Producers]. ICF [Distributor], 2012.
4. Institut National de la Statistique (INS) [Cameroon] et ICF. 2020. *Enquête Démographique et de Santé du Cameroun 2018* [Dataset]. CMMR71.DTA. Yaoundé, Cameroun et Rockville, Maryland, USA: INS et ICF [Producers]. ICF [Distributor], 2020.
5. Ouagadjio, Bandoumal, Kostelngar Nodjimadji, Tchobkréo Bagamla, Riradjim Madnodji, Joël Sibaye Tokindang, Ningam Ngakoutou, Joël Nodjimbatem Ngoniri, Caman Bédaou, Donato Koyalta, Bernard Barrère, Monique Barrère. 2004. *Enquête Démographique et de Santé Tchad 2004* [Dataset]. TDMR41.DTA. Calverton, Maryland, U.S.A.: INSEED et ORC Macro [Producers]. ICF [Distributor], 2004.
6. Institut National de la Statistique, des Études Économiques et Démographiques (INSEED) [Chad], Ministère de la Santé Publique (MSP) [Chad] et ICF International, 2014-2015. *Enquête Démographique et de Santé et à Indicateurs Multiples (EDS-MICS 2014-2015)* [Dataset]. TDMR71.DTA. Rockville, Maryland, USA: INSEED, MSP et ICF International [Producers]. ICF [Distributor], 2014-2015.
7. Centre Nationale de la Statistique et des Études Économiques (CNSEE) [Congo] et ICF International, 2013. *Enquête Démographique et de Santé du Congo (EDSC-II) 2011-2012* [Dataset]. CGMR61.DTA*.* Calverton, Maryland, USA: CNSEE et ICF International [Producers]. ICF [Distributor], 2013.
8. Ministère du Plan et Suivi de la Mise en œuvre de la Révolution de la Modernité (MPSMRM) [Democratic Republic of Congo], Ministère de la Santé Publique (MSP) [Democratic Republic of Congo] et ICF International, 2014. *Enquête Démographique et de Santé en République Démocratique du Congo 2013-2014* [Dataset]*.* CDMR61.DTA. Rockville, Maryland, USA: MPSMRM, MSP et ICF International [Producers]. ICF [Distributor], 2014.
9. Direction Générale de la Statistique et des Études Économiques (DGSEE) [Gabon] et ORC Macro. 2001. *Enquête Démographique et de Santé Gabon 2000* [Dataset]. GAMR41.DTA. Calverton, Maryland: Direction Générale de la Satistique et des Études Économiques, et Fonds des Nations Unies pour la Populations, et ORC Macro [Producers]. ICF [Distributor], 2001.
10. Direction Générale de la Statistique (DGS) [Gabon] et ICF International. 2013. *Enquête Démographique et de Santé du Gabon 2012* [Dataset]. GAMR61.DTA. Calverton, Maryland, et Libreville, Gabon: DGS et ICF International [Producers]. ICF [Distributor], 2013.
11. Institut National de la Statistique et de l’Analyse Économique (INSAE) [Benin] et ICF International, 2013. *Enquête Démographique et de Santé du Bénin 2011-2012* [Dataset]. BJMR61.DTA. Calverton, Maryland, USA: INSAE et ICF International [Producers]. ICF [Distributor], 2013.
12. Institut National de la Statistique et de l’Analyse Économique (INSAE) [Benin] et ICF. 2019. *Enquête Démographique et de Santé au Bénin, 2017-2018* [Dataset]. BJMR71.DTA. Cotonou, Bénin et Rockville, Maryland, USA: INSAE et ICF [Producers]. ICF [Distributor], 2019.
13. Institut National de la Statistique et de la Démographie (INSD) [Burkina Faso] et ICF International, 2012. *Enquête Démographique et de Santé et à Indicateurs Multiples du Burkina Faso 2010* [Dataset]. BFMR62.DTA*.* Calverton, Maryland, USA: INSD et ICF International [Producers]. ICF [Distributor], 2012.
14. Institut National de la Statistique (INS) [Côte d’Ivoire] et ICF International. 2012. *Enquête Démographique et de Santé et à Indicateurs Multiples de Côte d’Ivoire 2011-2012* [Dataset]. CIMR62.DTA. Calverton, Maryland, USA: INS et ICF International [Producers]. ICF [Distributor], 2012.
15. The Gambia Bureau of Statistics (GBOS) and ICF International. 2014. *The Gambia Demographic and Health Survey 2013* [Dataset]. GMMR61.DTA*.* Banjul, The Gambia, and Rockville, Maryland, USA: GBOS and ICF International [Producers]. ICF [Distributor], 2014.
16. Ghana Statistical Service (GSS), Noguchi Memorial Institute for Medical Research (NMIMR) [Ghana], and ORC Macro. 2004. *Ghana Demographic and Health Survey 2003* [Dataset]. GHMR4B.DTA. Calverton, Maryland: GSS, NMIMR, and ORC Macro [Producers]. ICF [Distributor], 2004.
17. Ghana Statistical Service (GSS), Ghana Health Service (GHS), and ICF International. 2015. *Ghana Demographic and Health Survey 2014* [Dataset]. GHMR71.DTA. Rockville, Maryland, USA: GSS, GHS, and ICF International [Producers]. ICF [Distributor], 2015.
18. Direction Nationale de la Statistique (DNS) [Guinea] et ORC Macro. 2006. *Enquête Démographique et de Santé, Guinée 2005* [Dataset]. GNMR52.DTA*.* Calverton, Maryland, U.S.A.: DNS et ORC Macro [Producers]. ICF [Distributor], 2006.
19. Institut National de la Statistique (INS) [Guinea] et ICF International. 2013. *Enquête Démographique et de Santé et à Indicateurs Multiples 2012* [Dataset]. GNMR62.DTA. Conakry, Guinée, et Calverton, Maryland, USA: INS et ICF [Producers]. ICF [Distributor], 2013.
20. Institut National de la Statistique (INS) [Guinea] et ICF. 2018. *Enquête Démographique et de Santé en Guinée 2018* [Dataset] GNMR71.DTA. Conakry, Guinée, et Rockville, Maryland, USA: INS et ICF [Producers]. ICF [Distributor], 2018.
21. Liberia Institute of Statistics and Geo-Information Services (LISGIS), Ministry of Health and Social Welfare [Liberia], National AIDS Control Program [Liberia], and ICF International. 2014. *Liberia Demographic and Health Survey 2013* [Dataset]. LBMR6A.DTA*.* Monrovia, Liberia: Liberia Institute of Statistics and GeoInformation Services (LISGIS) and ICF International [Producers]. ICF [Distributor], 2014.
22. Cellule de Planification et de Statistique (CPS/SSDSPF) [Mali], Institut National de la Statistique (INSTAT/MPATP) [Mali], INFO-STAT et ICF International. 2014. *Enquête Démographique et de Santé au Mali 2012-2013* [Dataset]. MLMR6A.DTA*.* Rockville, Maryland, USA: CPS, INSTAT, INFO-STAT et ICF International [Producers]. ICF [Distributor], 2014.
23. Institut National de la Statistique (INSTAT) [Mali], Cellule de Planification et de Statistique Secteur Santé-Développement Social et Promotion de la Famille (CPS/SS-DS-PF) [Mali] et ICF. 2019. *Enquête Démographique et de Santé au Mali 2018* [Dataset]. MLMR7A.DTA. Bamako, Mali et Rockville, Maryland, USA: INSTAT, CPS/SS-DS-PF et ICF [Producers]. ICF [Distributor], 2019.
24. Institut National de la Statistique (INS) [Niger] et ICF International. 2013. *Enquête Démographique et de Santé et à Indicateurs Multiples du Niger 2012* [Dataset]. NIMR61.DTA. Calverton, Maryland, USA: INS et ICF International [Producers]. ICF [Distributor], 2013.
25. National Population Commission (NPC) [Nigeria] and ORC Macro. 2004. *Nigeria Demographic and Health Survey 2003* [Dataset]. NGMR4A.DTA. Calverton, Maryland: National Population Commission and ORC Macro [Producers]. ICF [Distributor], 2004.
26. National Population Commission (NPC) [Nigeria] and ICF International. 2014. *Nigeria Demographic and Health Survey 2013* [Dataset]. NGMR6A.DTA. Abuja, Nigeria, and Rockville, Maryland, USA: NPC and ICF International [Producers]. ICF [Distributor], 2014.
27. National Population Commission (NPC) [Nigeria] and ICF. 2019. *Nigeria Demographic and Health Survey 2018* [Dataset]. NGMR7A.DTA*.* Abuja, Nigeria, and Rockville, Maryland, USA: NPC and ICF [Producers]. ICF [Distributor], 2019.
28. Ndiaye, Salif, et Mohamed Ayad. 2006. *Enquête Démographique et de Santé au Sénégal 2005* [Dataset]. SNMR4A.DTA*.* Calverton, Maryland, USA: Centre de Recherche pour le Développement Humain [Sénégal] et ORC Macro [Producers]. ICF [Distributor], 2006.
29. Agence Nationale de la Statistique et de la Démographie (ANSD) [Sénégal], et ICF International. 2012. *Enquête Démographique et de Santé à Indicateurs Multiples au Sénégal (EDS-MICS) 2010-2011* [Dataset]. SNMR61.DTA. Calverton, Maryland, USA: ANSD et ICF International [Producers]. ICF [Distributor], 2012.
30. Agence Nationale de la Statistique et de la Démographie (ANSD) [Sénégal], et ICF International. 2015. *Sénégal : Enquête Démographique et de Santé Continue (EDS-Continue 2012-14), Rapport Régional* [Dataset]. SNMR70.DTA. Rockville, Maryland, USA: ANSD et ICF International [Producers]. ICF [Distributor], 2015.
31. Agence Nationale de la Statistique et de la Démographie (ANSD) [Sénégal], et ICF. 2016*. Sénégal : Enquête Démographique et de Santé Continue (EDS-Continue 2015)* [Dataset]. SNMR7H.DTA. Rockville, Maryland, USA: ANSD et ICF [Producers]. ICF [Distributor], 2016.
32. Agence Nationale de la Statistique et de la Démographie (ANSD) [Sénégal], et ICF. 2017. *Sénégal : Enquête Démographique et de Santé Continue (EDS-Continue 2016)* [Dataset]. SNMR7I.DTA. Rockville, Maryland, USA: ANSD et ICF [Producers]. ICF [Distributor], 2017.
33. Agence Nationale de la Statistique et de la Démographie (ANSD) [Sénégal], et ICF. 2018. *Sénégal : Enquête Démographique et de Santé Continue (EDS-Continue 2017)* [Dataset]. SNMR7Z.DTA. Rockville, Maryland, USA: ANSD et ICF [Producers]. ICF [Distributor], 2018.
34. Agence Nationale de la Statistique et de la Démographie (ANSD) [Sénégal], et ICF. 2018. *Sénégal : Enquête Démographique et de Santé Continue (EDS-Continue 2018)* [Dataset]. SNMR81.DTA. Rockville, Maryland, USA: ANSD et ICF [Producers]. ICF [Distributor], 2018.
35. Agence Nationale de la Statistique et de la Démographie (ANSD) [Sénégal], et ICF. 2019. *Sénégal : Enquête Démographique et de Santé Continue (EDS-Continue 2019)* [Dataset]*.* SNMR8B.DTA. Rockville, Maryland, USA : ANSD et ICF [Producers]. ICF [Distributor], 2019.
36. Statistics Sierra Leone (SSL) and ICF International. 2014. *Sierra Leone Demographic and Health Survey 2013* [Dataset]. SLMR61.DTA. Freetown, Sierra Leone and Rockville, Maryland, USA: SSL and ICF International [Producers]. ICF [Distributor], 2014.
37. Statistics Sierra Leone (Stats SL) and ICF. 2020. *Sierra Leone Demographic and Health Survey 2019* [Dataset]. SLMR7A.DTA*.* Freetown, Sierra Leone, and Rockville, Maryland, USA: Stats SL and ICF [Producers]. ICF [Distributor], 2020.
38. Ministère de la Planification, du Développement et de l’Aménagement du Territoire (MPDAT) [Togo], Ministère de la Santé (MS) [Togo] et ICF International, 2015. *Enquête Démographique et de Santé au Togo 2013-2014* [Dataset]. TGMR61.DTA. Rockville, Maryland, USA: MPDAT, MS et ICF International [Producers]. ICF [Distributor], 2015.
39. Institut de Statistiques et d’Études Économiques du Burundi (ISTEEBU), Ministère de la Santé Publique et de la Lutte contre le Sida [Burundi] (MSPLS), et ICF International. 2012. *Enquête Démographique et de Santé Burundi 2010* [Dataset]*.* BUMR61.DTA. Bujumbura, Burundi: ISTEEBU, MSPLS, et ICF International [Producers]. ICF [Distributor], 2012.
40. Ministère à la Présidence chargé de la Bonne Gouvernance et du Plan [Burundi] (MPBGP), Ministère de la Santé Publique et de la Lutte contre le Sida [Burundi] (MSPLS), Institut de Statistiques et d’Études Économiques du Burundi (ISTEEBU), et ICF. 2017. *Troisième Enquête Démographique et de Santé* [Dataset]*.* BUMR761.DTA. Bujumbura, Burundi: ISTEEBU, MSPLS, et ICF [Producers]. ICF [Distributor], 2017.
41. Direction Générale de la Statistique et de la Prospective (DGSP) et ICF International. 2014. *Enquête Démographique et de Santé et à Indicateurs Multiples aux Comores 2012* [Dataset]*.* KMMR61.DTA. Rockville, MD 20850, USA: DGSP et ICF International [Producers]. ICF [Distributor], 2014.
42. Central Statistical Authority [Ethiopia] and ORC Macro. 2001. *Ethiopia Demographic and Health Survey 2000* [Dataset]*.* ETMR41.DTA. Addis Ababa, Ethiopia and Calverton, Maryland, USA: Central Statistical Authority and ORC Macro [Producers]. ICF [Distributor], 2001.
43. Central Statistical Agency [Ethiopia] and ORC Macro. 2006. *Ethiopia Demographic and Health Survey 2005* [Dataset]*.* ETMR51.DTA. Addis Ababa, Ethiopia and Calverton, Maryland, USA: Central Statistical Agency and ORC Macro [Producers]. ICF [Distributor], 2006.
44. Central Statistical Agency [Ethiopia] and ICF International. 2012. *Ethiopia Demographic and Health Survey 2011* [Dataset]*.* ETMR61.DTA. Addis Ababa, Ethiopia and Calverton, Maryland, USA: Central Statistical Agency and ICF International [Producers]. ICF [Distributor], 2012.
45. Central Statistical Agency (CSA) [Ethiopia] and ICF. 2016. *Ethiopia Demographic and Health Survey 2016* [Dataset]*.* ETMR71.DTA. Addis Ababa, Ethiopia, and Rockville, Maryland, USA: CSA and ICF [Producers]. ICF [Distributor], 2016.
46. Central Bureau of Statistics (CBS) [Kenya], Ministry of Health (MOH) [Kenya], and ORC Macro. 2004. *Kenya Demographic and Health Survey 2003* [Dataset]*.* KEMR42.DTA. Calverton, Maryland: CBS, MOH, and ORC Macro [Producers]. ICF [Distributor], 2004.
47. National AIDS and STI Control Programme (NASCOP), Kenya. *Kenya AIDS Indicator Survey 2012: Final Report*. Nairobi, NASCOP. June 2014.
48. Kenya National Bureau of Statistics (NBS), Ministry of Health (MOH) [Kenya], and ICF International. 2015. *Kenya Demographic and Health Survey 2014* [Dataset]. KEMR72.DTA. Nairobi, Kenya, and Rockville, Maryland, USA: NBS, MOH, and ICF International [Producers]. ICF [Distributor], 2015.
49. Mariko, Soumaïla et Victor Rabeza. 2005. *Enquête de Base sur la Santé de la Reproduction et la Survie des Enfants dans les zones d’intervention USAID, à Madagascar - EBSRSE 2003-2004* [Dataset]. MDMR42.DTA*.* Calverton, Maryland, USA : INSTAT et ORC Macro [Producers]. ICF [Distributor], 2005.
50. National Statistical Office [Malawi] and ORC Macro. 2001. *Malawi Demographic and Health Survey 2000* [Dataset]. MWMR41.DTA*.* Zomba, Malawi and Calverton, Maryland, USA: National Statistical Office and ORC Macro [Producers]. ICF [Distributor], 2001.
51. National Statistical Office (NSO) [Malawi], and ORC Macro. 2005. *Malawi Demographic and Health Survey 2004* [Dataset]. MWMR4E.DTA*.* Calverton, Maryland: NSO and ORC Macro [Producers]. ICF [Distributor], 2005.
52. National Statistical Office (NSO) and ICF Macro. 2011. *Malawi Demographic and Health Survey 2010* [Dataset]. MWMR61.DTA*.* Zomba, Malawi, and Calverton, Maryland, USA: NSO and ICF Macro [Producers]. ICF [Distributor], 2011.
53. National Statistical Office (NSO) [Malawi] and ICF. 2017. *Malawi Demographic and Health Survey 2015-16* [Dataset]. MWMR7A.DTA. Zomba, Malawi, and Rockville, Maryland, USA. NSO and ICF [Producers]. ICF [Distributor], 2017.
54. Ministry of Health, Malawi. *Malawi Population-Based HIV Impact Assessment (MPHIA) 2015-2016: Final Report*. Lilongwe, Ministry of Health. October 2018.
55. Instituto Nacional de Estatística (INE) [Mozambique], Ministerio da Saude (MISAU) [Mozambique], e ORC Macro. 2005. *Moçambique Inquérito Demográfico e de Saúde 2003* [Dataset]*.* MZMR41.DTA. Maputo, Moçambique, and Calverton, Maryland: INE, MISAU, e ORC Macro [Producers]. ICF [Distributor], 2005.
56. Ministerio da Saude (MISAU), Instituto Nacional de Estatística (INE) e ICF International (ICFI), 2013. *Moçambique Inquérito Demográfico e de Saúde 2011* [Dataset]*.* MZMR62.DTA. Calverton, Maryland, USA: MISAU, INE e ICFI [Producers]. ICF [Distributor], 2013.
57. Ministério da Saúde (MISAU), Instituto Nacional de Estatística (INE), e ICF, 2015. *Inquérito de Indicadores de Imunização, Malária e HIV/SIDA em Moçambique 2015* [Dataset]*.* MZMR71.DTA. Maputo, Moçambique, and Rockville, Maryland, EUA: INS, INE, e ICF [Producers]. ICF [Distributor], 2015.
58. Office National de la Population (ONAPO) [Rwanda] et ORC Macro. 2001. *Enquête Démographique et de Santé, Rwanda 2000* [Dataset]*.* RWMR41.DTA. Kigali, Rwanda et Calverton, Maryland, USA : Ministère de la Santé, Office National de la Population et ORC Macro [Producers]. ICF [Distributor], 2001.
59. Institut National de la Statistique du Rwanda (INSR) and ORC Macro. 2006. *Rwanda Demographic and Health Survey 2005* [Dataset]*.* RWMR53.DTA. Calverton, Maryland, U.S.A.: INSR and ORC Macro [Producers]. ICF [Distributor], 2006.
60. National Institute of Statistics of Rwanda (NISR) [Rwanda], Ministry of Health (MOH) [Rwanda], and ICF International. 2012. *Rwanda Demographic and Health Survey 2010* [Dataset]*.* RWMR61.DTA. Calverton, Maryland, USA: NISR, MOH, and ICF International [Producers]. ICF [Distributor], 2012.
61. National Institute of Statistics of Rwanda (NISR) [Rwanda], Ministry of Health (MOH) [Rwanda], and ICF International. 2015. *Rwanda Demographic and Health Survey 2014-15* [Dataset]*.* RWMR70.DTA. Rockville, Maryland, USA: NISR, MOH, and ICF International [Producers]. ICF [Distributor], 2015.
62. National Bureau of Statistics (NBS) [Tanzania] and ORC Macro. 2005. *Tanzania Demographic and Health Survey 2004-05* [Dataset]*.* TZMR4I.DTA. Dar es Salaam, Tanzania: National Bureau of Statistics and ORC Macro [Producers]. ICF [Distributor], 2005.
63. National Bureau of Statistics (NBS) [Tanzania] and ICF Macro. 2011. *Tanzania Demographic and Health Survey 2010* [Dataset]*.* TZMR61.DTA. Dar es Salaam, Tanzania: NBS and ICF Macro [Producers]. ICF [Distributor], 2011.
64. Tanzania Commission for AIDS (TACAIDS), Zanzibar AIDS Commission (ZAC), National Bureau of Statistics (NBS), Office of the Chief Government Statistician (OCGS), and ICF International 2013. *Tanzania HIV/AIDS and Malaria Indicator Survey 2011-12* [Dataset]*.* TZIR6A.DTA. Dar es Salaam, Tanzania: TACAIDS, ZAC, NBS, OCGS, and ICF International [Producers]. ICF [Distributor], 2013.
65. Uganda Bureau of Statistics (UBOS) and ORC Macro. 2001. *Uganda Demographic and Health Survey 2000-2001* [Dataset]*.* UGMR41.DTA. Calverton, Maryland, USA: UBOS and ORC Macro [Producers]. ICF [Distributor], 2001.
66. Uganda Bureau of Statistics (UBOS) and ICF International Inc. 2012. *Uganda Demographic and Health Survey 2011* [Dataset]*.* UGMR61.DTA. Kampala, Uganda: UBOS and Calverton, Maryland: ICF International Inc [Producers]. ICF [Distributor], 2012.
67. Uganda Bureau of Statistics (UBOS) and ICF. 2018. *Uganda Demographic and Health Survey 2016* [Dataset]*.* UGMR7B.DTA. Kampala, Uganda and Rockville, Maryland, USA: UBOS and ICF [Producers]. ICF [Distributor], 2018.
68. Central Statistical Office [Zambia], Central Board of Health [Zambia], and ORC Macro. 2003. *Zambia Demographic and Health Survey 2001-2002* [Dataset]*.* ZMMR41.DTA. Calverton, Maryland, USA: Central Statistical Office, Central Board of Health, and ORC Macro [Producers]. ICF [Distributor], 2003.
69. Central Statistical Office (CSO) [Zambia], Ministry of Health (MOH) [Zambia], and ICF International. 2014. *Zambia Demographic and Health Survey 2013-14* [Dataset]*.* ZMMR61.DTA. Rockville, Maryland, USA: Central Statistical Office, Ministry of Health, and ICF International [Producers]. ICF [Distributor], 2014.
70. Ministry of Health, Zambia. *Zambia Population-based HIV Impact Assessment (ZAMPHIA) 2016: Final Report*. Lusaka, Ministry of Health. February 2019.
71. Zambia Statistics Agency, Ministry of Health (MOH) [Zambia], and ICF. 2019. *Zambia Demographic and Health Survey 2018* [Dataset]*.* ZMMR71.DTA. Lusaka, Zambia, and Rockville, Maryland, USA: Zambia Statistics Agency, Ministry of Health, and ICF [Producers]. ICF [Distributor], 2019.
72. Zimbabwe National Statistics Agency (ZIMSTAT) and ICF International. 2012. *Zimbabwe Demographic and Health Survey 2010-11* [Dataset]*.* ZWMR62.DTA. Calverton, Maryland: ZIMSTAT and ICF International Inc [Producers]. ICF [Distributor], 2012.
73. Zimbabwe National Statistics Agency and ICF International. 2016. *Zimbabwe Demographic and Health Survey 2015* [Dataset]*.* ZWMR72.DTA. Rockville, Maryland, USA: Zimbabwe National Statistics Agency (ZIMSTAT) and ICF International [Producers]. ICF [Distributor], 2016.
74. Government of the Kingdom of Eswatini. *Swaziland HIV Incidence Measurement Survey 2 (SHIMS2) 2016-2017. Final Report*. Mbabane: Government of the Kingdom of Eswatini; April 2019.
75. Ministry of Health and Social Welfare (MOHSW) [Lesotho], Bureau of Statistics (BOS) [Lesotho], and ORC Macro. 2005. *Lesotho Demographic and Health Survey 2004* [Dataset]. LSMR41.DTA. Calverton, Maryland: MOH, BOS, and ORC Macro [Producers]. ICF [Distributor], 2005.
76. Ministry of Health and Social Welfare (MOHSW) [Lesotho] and ICF Macro. 2010. *Lesotho Demographic and Health Survey 2009* [Dataset]. LSMR61.DTA. Maseru, Lesotho: MOHSW and ICF Macro [Producers]. ICF [Distributor], 2010.
77. Ministry of Health [Lesotho] and ICF International. 2016. *Lesotho Demographic and Health Survey 2014* [Dataset]. LSMR71.DTA. Maseru, Lesotho: Ministry of Health and ICF International [Producers]. ICF [Distributor], 2016
78. Ministry of Health and Social Services (MOHSS) [Namibia]. 2003. *Namibia Demographic and Health Survey 2000* [Dataset]. NMMR41.DTA.Windhoek, Namibia: MOHSS [Producers]. ICF [Distributor], 2003.
79. The Nambia Ministry of Health and Social Services (MoHSS) and ICF International. 2014. *The Namibia Demographic and Health Survey 2013* [Dataset]. NMMR61.DTA. Windhoek, Namibia, and Rockville, Maryland, USA: MoHSS and ICF International [Producers]. ICF [Distributor], 2014.
80. Centers for Disease Control and Prevention (CDC), Global Clinical and Viral Laboratory (South Africa), Human Sciences Research Council, National Institute for Communicable Diseases (South Africa), South African Medical Research Council, University of Cape Town. *South Africa National HIV Prevalence, Incidence, and Behavior Survey 2011-2012*. Pretoria, South Africa: Human Sciences Research Council, 2016.
81. National Department of Health (NDoH), Statistics South Africa (Stats SA), South African Medical Research Council (SAMRC), and ICF. 2019. *South Africa Demographic and Health Survey 2016* [Dataset]. ZAMR71.DTA. Pretoria, South Africa, and Rockville, Maryland, USA: NDoH, Stats SA, SAMRC, and ICF [Producers]. ICF [Distributor], 2019.
82. Centers for Disease Control and Prevention (CDC), Human Sciences Research Council, National Institute for Communicable Diseases (South Africa), South African Medical Research Council. *South Africa National HIV Prevalence, Incidence, Behavior and Communication Survey 2017.*
83. Ministry of Health, Lesotho, Centers for Disease Control and Prevention (CDC), and ICAP at Columbia University. Lesotho Population-based HIV Impact Assessment (LePHIA) 2016-2017: Final Report. Maseru, Lesotho, Atlanta, Georgia, and New York, New York, USA: Ministry of Health, CDC, and ICAP, September 2019.
84. Ministère de la Santé et de l’Hygiène Publique (MSHP). Côte d’Ivoire Population-Based HIV Impact Assessment (CIPHIA) 2017-2018: Final Report. Abidjan: MSHP; March 2021.
85. Ministry of Health and Social Services (MoHSS), Namibia. Namibia Population-based HIV Impact Assessment (NAMPHIA) 2017: Final Report. Windhoek: MoHSS, Namibia; November, 2019.
86. Liberia Institute of Statistics and Geo-Information Services (LISGIS), Ministry of Health [Liberia], and ICF. 2021. *Liberia Demographic and Health Survey 2019-20* [Dataset]*.* LBMR7A.DTA. Monrovia, Liberia and Rockville, Maryland, USA: Liberia Institute of Statistics and Geo-Information Services (LISGIS), Ministry of Health, and ICF [Producers]. ICF [Distributor], 2021.
87. Gambia Bureau of Statistics (GBoS) and ICF. 2021. *The Gambia Demographic and Health Survey 2019-20* [Dataset]. GMMR81DTA. Banjul, The Gambia and Rockville, Maryland, USA: GBoS and ICF [Producers]. ICF [Distributor], 2021.
