# Supplementary material for: Population sizes, HIV prevalence, and HIV prevention among men who paid for sex in sub-Saharan Africa (2000–2020): A meta-analysis of 87 population-based surveys
Source: PLoS Med. 2022 Jan 25;19(1):e1003861. doi: 10.1371/journal.pmed.1003861 (PMC8789156; doi:10.1371/journal.pmed.1003861)
Supplement: Attachment — Submitted filename: hodgins.pdf [file pmed.1003861.s002.pdf]

This paper reports a meta–analysis of existing surveys on men who paid for sex in sub–Saharan Africa.

I was asked for a statistical report and I interpret that to include all aspects of the design and conduct of the study.

Points of detail

Page 2 'Paying for sex is prevalent across SSA [. . . ']. Well, yes, but presumably elsewhere too so I wonder whether a careless reader might interpret this as stigmatising the men in SSA? If the authors are happy with the wording then fine but otherwise perhaps replace 'across SSA' with 'in the countries studied'? Obviously I know the authors are not trying to stigmatise anyone.

Page 5 Were the pooled proportions meta–analysed untransformed? In the meta–regression we read later that a transformation was applied.

Page 5 In an ideal world number of partners would be analysed on the log scale, being a count, or some other transform. Is the data not available to do that?

Page 6 The light blue dots did not stand out very well as printed here (I do have normal colour vision). Is there some other choice?

Table S4 How was the formal test for time carried out? The table makes it appear that just two years were compared but that seems both unlikely and sub–optimal.

Figure S3 Should the caption read 'three or more surveys'? I would have to say that even for those with more than that I would not be inclined to place much reliance in individual slopes. Even for Senegal the line must be influenced by 2005 and some of the others are subject to high leverage points too.

Figure S6 Same point about number of points as for S3. This one does look much better behaved though

The introduction has a focus on female sex workers but looking at Table S1 some of the surveys specifically ask about sex with women (French: *quelq'une* versus *quelqu'un*; Portuguese: *com uma mulher* versus *com alguém*). Is this a limitation?

Points of more substance

Clustering

There are multiple estimates from some countries. This seems likely to have introduced a lack of independence which is one of the crucial assumptions of meta–analysis. I see the authors used metafor so they might like to investigate the robust() command or, following the suggestion in its help page, look at the clubSandwich package which can be used with metafor.

Heterogeneity

I see the authors have, wisely in my view, made no attempt to say much about the expected massive heterogeneity. I wonder though whether there is a case here for more extensive use of prediction intervals which would give us limits for the next study in addition to the current confidence intervals which tell us about the sampling error of the underlying true effect. The advantages of prediction intervals have been described by Riley et al. (2011).

Summary

No major concerns but some points to clear up.

Michael Dewey

References

R D Riley, J P T Higgins, and J J Deeks. Interpretation of random effects meta–analyses. *British Medical Journal*, 342:964–967, 2011.
