# Supplementary material for: Population sizes, HIV prevalence, and HIV prevention among men who paid for sex in sub-Saharan Africa (2000–2020): A meta-analysis of 87 population-based surveys
Source: PLoS Med. 2022 Jan 25;19(1):e1003861. doi: 10.1371/journal.pmed.1003861 (PMC8789156; doi:10.1371/journal.pmed.1003861)
Supplement: Attachment — Submitted filename: Response to reviewers.pdf [file pmed.1003861.s003.pdf]

Dear Dr Richard Turner,

We are pleased to submit a revised version of our manuscript titled "*HIV prevalence, population sizes, and HIV prevention among men who paid for sex in sub-Saharan Africa: a meta-analysis of 87 population-based surveys (2000-2020)".*

We thank the reviewers for their insightful feedback on our paper and are grateful to address their constructive comments. We provide a point-by-point response in the following pages and have incorporated suggestions into the paper to improve clarity. The main changes are outlined below:

- Added 2 new DHS and 3 new PHIA surveys that have just been released. These surveys improved our estimates of ART coverage and viral load suppression.
- Correction to the denominators of the Cameroon and Chad 2004 DHS.
- Considered the sampling strata to obtain our estimates.
- Further investigated heterogeneity by examining prevalence of paid sex by age group and urban/rural strata.
- Prediction intervals were added to the pooled estimates, as suggested by the first reviewer.
- The MOOSE checklist has been completed.
- A more thorough discussion of implications of our study for HIV prevention and control.

We are confident that the manuscript has been significantly improved as a result of the reviewers' thorough assessment of our work.

Sincerely,

Caroline Hodgins, James Stannah, Salome Kuchukhidze, and Mathieu Maheu-Giroux, on behalf of all co-authors

Reviewer #1:
This paper reports a meta-analysis of existing surveys on men who paid for sex in sub-Saharan Africa.
I was asked for a statistical report and I interpret that to include all aspects of the design and conduct of the study.

Points of detail
*1. **Page 2** 'Paying for sex is prevalent across SSA [. . . ']. Well, yes, but presumably elsewhere too so I wonder whether a careless reader might interpret this as stigmatising the men in SSA? If the authors are happy with the wording then fine but otherwise perhaps replace 'across SSA' with 'in the countries studies'? Obviously I know the authors are not trying to stigmatise anyone.*

> **Response:** We agree with the reviewer and have changed the wording of the abstract on page 2 to read as follows:
>
> *'Paying for sex is prevalent and men who ever paid for sex were 50% more likely to be living with HIV compared to other men in these 35 countries.'*
>
> However, we are confident that our results are representative of men in the region. The 35 included countries represent more than 95% of all men aged 15-54 years in sub-Saharan Africa.

*2. **Page 5** Were the pooled proportions meta-analysed untransformed? In the meta-regression we read later that a transformation was applied.*

> **Response:** The pooled proportions were meta-analyzed using a logit transformation. We have added a sentence in the Methods section on page 7 to make this clearer:
>
> '*Meta-analyses were performed on logit-transformed proportions and log-transformed prevalence ratios.'*

*3. **Page 5** In an ideal world number of partners would be analysed on the log scale, being a count, or some other transform. Is the data not available to do that?*

> **Response:** We have adjusted our methods to analyze log-transformed lifetime number of partners and have edited the Methods section on page 7 to reflect this change. These results were minimally affected by the type of transformation (12.0 with log-transformation vs. 12.7 partners on the original scale).
>
> *'We calculated the following estimands . . . log-transformed mean and ratio of means of lifetime sexual partners for men who paid for sex and those who had not.'*

4. ***Page 6*** *The light blue dots did not stand out very well as printed here (I do have normal colour vision). Is there some other choice?*

> **Response:** We are grateful for the reviewer's suggestion and have changed the color of these dots in Figure 1 to a darker blue, to ensure they are clear when printed, as shown below. We have also clarified the legend.

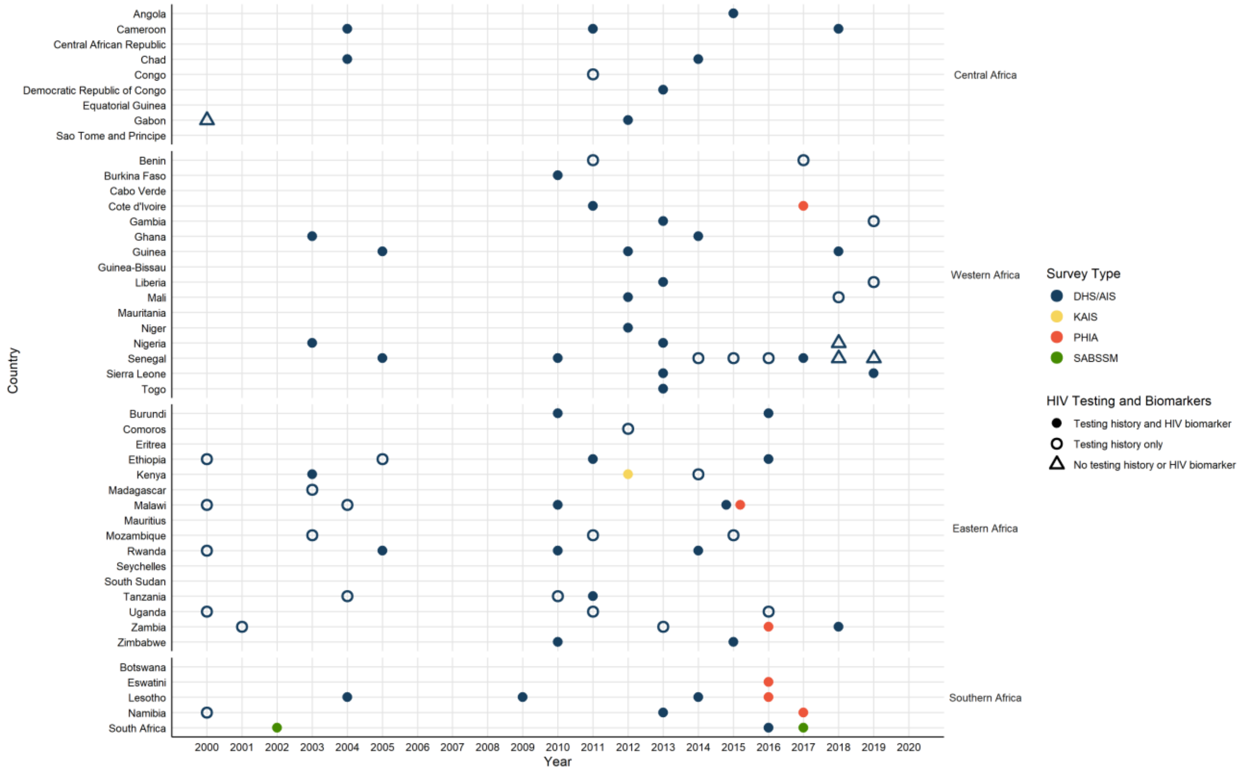

5. ***Table S4*** *How was the formal test for time carried out? The table makes it appear that just two years were compared but that seems both unlikely and sub-optimal.*

> **Response:** To assess variation in survey estimates of outcomes over time, we performed univariable meta-regression with survey year included as a continuous predictor. Using these models, we predicted outcomes for the years 2010 and 2020, which we present in Table S4 (now called Table S5) on page 18 of the supplement. We thank the reviewer for highlighting the confusion caused by presenting just these two predictions and have edited Table S5 to additionally include the slope and 95% confidence interval of the linear trend for each outcome.

6. ***Figure S3*** *Should the caption read 'three or more surveys'? I would have to say that even for those with more than that I would not be inclined to place much reliance in individual slopes. Even for Senegal the line must be influenced by 2005 and some of the others are subject to high leverage points too.*

Response: We thank the reviewer for pointing this out. We have corrected the wording of the caption for Figure S3 on page 20 of the supplement to read '*three or more surveys*'. We agree with the reviewer that some of the slopes are heavily influenced by one survey, and therefore we have not attempted to draw strong conclusions from these plots. We have included them in the supplementary materials to visualize within-country trends in paid sex and condom use over time.

*7. **Figure S6** Same point about number of points as for S3. This one does look much better behaved though.*

Response: We have corrected the caption of Figure S6 on page 24 of the supplement to read '*three or more surveys*'.

8. The introduction has a focus on female sex workers but looking at Table S1 some of the surveys specifically ask about sex with women (French: quelq'une versus quelqu'un; Portuguese: com uma mulher versus com alguém). Is this a limitation?

Response: We agree with the reviewer that most surveys did not specifically refer to paid sex with a woman. The survey instruments do not collect information on the sex of commercial partners, or on whether clients have sex with women and/or men. However, globally, UNAIDS estimates that approximately 1.9% of men also have sex with other men (UNAIDS 2020). In Western and Central Africa, where the majority of men included in our analyses were surveyed, this estimate ranges from 0.45% to 1.5% (UNAIDS and WHO 2020). As such, the number of male clients included in our analyses who exclusively pay for sex with men is likely to be small and unlikely to affect the conclusions of our analyses. Further, men who paid for sex with men would also be at higher risk of HIV acquisition and transmission. Our introduction focused on female sex workers because this group is believed to form the majority of individuals involved in sex work. However, for precision, we have modified the abstract and introduction to refer more generally to "*sex workers*" rather than "*female sex workers*". Furthermore, we have added to the discussion on page 19 to address the potential limitation raised by this reviewer:

Third, most surveys do not specify the paid partner's gender when asking about paid sex and we cannot be certain that all men in our analytical sample have paid for sex with a woman. However, the proportion of men who have sex with men in this region is estimated to be less than 2% [50].

We have also clarified in the Supplementary Materials on page 1 that only nine surveys specified paid sex with a woman, while the others did not specify partner gender.

References:

UNAIDS. Seizing the moment: tackling entrenched inequalities to end epidemics. Global AIDS update. Geneva: UNAIDS; 2020. https://www.unaids.org/en/resources/documents/2020/global-aids-report

UNAIDS and WHO. Recommended population size estimates of men who have sex with men. 2020.

https://www.unaids.org/sites/default/files/media_asset/2020-recommended-population-size-estimates-of-men-who-have-sex-with-men_en.pdf

Points of more substance
Clustering
1. There are multiple estimates from some countries. This seems likely to have introduced a lack of independence which is one of the crucial assumptions of meta-analysis. I see the authors used metafor so they might like to investigate the robust() command or, following the suggestion in its help page, look at the clubSandwich package which can be used with metafor.

Response: We thank the reviewer for this suggestion. We have updated this analysis as suggested using the *robust()* command, to account for clustering of surveys within countries. As expected, the point estimates are unaffected, but the confidence intervals are slightly larger.

Heterogeneity
2. I see the authors have, wisely in my view, made no attempt to say much about the expected massive heterogeneity. I wonder though whether there is a case here for a more extensive use of prediction intervals which would give us limits for the next study in addition to the current confidence intervals which tell us about the sampling error of the underlying true effect. The advantages of prediction intervals have been described by Riley et al. (2011).

Response: Indeed, a high level of heterogeneity was expected given the research questions addressed by this study. We agree with the reviewer, and we have now included prediction intervals in Table S2 (now called Table S3) on pages 13-16 of the supplement.

Summary
No major concerns but some points to clear up.

Reviewer #2:
This is an excellent study that is adding to the small number of evidence on the clients of sex workers.
However, the authors did not address many of the issues they have highlighted. Using your critical minds and wealth of knowledge, what recommendations/ suggestions to you have for the public health authorities, the FSWs or to even clients of sex workers?

Response: We thank the reviewer for the review and the opportunity to elaborate and expand upon potential recommendations for public health authorities based on the results of our analyses. When discussing our results, we have attempted not to overreach with strong one-size-fits-all recommendations.

Our main recommendation is that greater attention from public health authorities should be devoted to men who pay for sex. Specifically, we called for a formal recognition of men who pay for sex as a priority population. Tailored interventions should be developed specifically for this group.

We argue this in our conclusions on page 20 by saying that "*men who pay for sex continue to constitute a distinct population subgroup at high risk of HIV acquisition and transmission*", which leads us to recommend that "*they should be recognized as a priority population for HIV prevention*".

To further address the reviewer's comment, we have added a further recommendation on page 18 that "*distribution of HIV self-tests to FSW, who can then distribute the tests to peers, clients, and partners, may further improve knowledge of status among men who pay for sex*". This has been done successfully in three Western African countries as part of the ATLAS project (Rouveau, 2021), in which HIV self-tests were distributed to female sex workers, who then distributed them to their peers, clients, and other partners. Involving public health authorities in the distribution of HIV self-tests will be important for increasing HIV testing and improving linkage into care for sex workers, male clients, and their sexual networks.

We have also added a further recommendation on page 18 of our Discussion: "*treatment access can be facilitated by services targeted to men who are more likely to frequent sex workers, such as migrant labourers, long-distance truck drivers, mine workers, and other men who travel for work [41]*".

References:

Rouveau N, Ky-Zerbo O, Boye S, Fotso AS, d'Elbée M, Maheu-Giroux M, et al. Describing, analysing and understanding the effects of the introduction of HIV self-testing in West Africa through the ATLAS programme in Côte d'Ivoire, Mali and Senegal. BMC Public Health 2021; 21: 181.

Baleta A. Lives on the line: sex work in sub-Saharan Africa. Lancet 2015; 385: e1-e2.

It would have been interesting to know:
1. their policy recommendations for public health authorities?

Response: As argued in the preceding paragraph, recommending a very specific one-size-fits-all approach to address the unmet prevention needs of men who pay for sex is difficult. There are many sex work typologies and local public health authorities should consider their local epidemic contexts to implement appropriate interventions. Our goal for this study is to draw attention to the priority population of men who pay for sex. We believe that such attention will renew interest in HIV prevention for this population group specifically, and share the burden of HIV prevention during sex work between clients and sex workers. If left behind, men who pay for sex will become increasingly important as the HIV epidemic in non-key populations decreases. Therefore, we recommended that public health authorities recognize men who pay for sex as a distinct population at risk of acquiring and transmitting HIV, and that they require targeted interventions. Implicit in this recommendation is that HIV surveillance should disaggregate epidemiological statistics by this population.

2. any recommendations for public health authorities using sex workers are index clients?

Response: We are hesitant to recommend the use of sex workers as index clients by public health authorities. This would require sex workers to disclose the identity of their male clients which may put sex workers at risk of violence, loss of sex work income, or both. Furthermore, index testing of sex workers places the responsibility to prevent HIV transmission during sex work solely on them. Overall, we feel that interventions should not require sex workers to disclose the identity of their clients, which we have added to page 18 of our Discussion.

Instead, we recommend on page 18 that public health authorities be involved in the distribution of HIV self tests among sex workers and their clients to increase knowledge of status among those living with HIV. Secondary distribution of self-test kits by sex workers may make it possible to engage hard-to-reach key populations of men who pay for sex without requiring partner disclosure. These interventions should also be paired with sex worker empowerment and education campaigns focused on how to approach negotiation of HIV self-testing and condom use.

3. what prevention approaches to tackle the high HIV prevalence in Western Africa versus the Southern?

Response: To clarify, our results show that HIV prevalence among men who pay for sex is lower in Western Africa than Southern Africa, but that the HIV prevalence ratio comparing men who do, and do not pay for sex, is higher. This suggests that in Western Africa, men who pay for sex are more likely to be living with HIV compared to men who do not pay for sex, than men in Southern Africa, but the overall HIV prevalence among all men is lower. Consequently, it may be more cost-effective to prioritize interventions to key and vulnerable populations in Western Africa, compared to Southern Africa where overall HIV prevalence is higher. Irrespective of the context, we recommend prevention approaches that are specifically focused on the unmet needs of men who pay for sex.

We have added the following to the Discussion section on page 18 to clarify this:

'Although HIV prevalence was lowest in Western Africa, this region had the highest HIV prevalence ratio comparing men who pay for sex with those who do not. In these settings, adding interventions that focus on the unmet prevention needs of men who pay for sex may be more cost-effective than those targeting the general population.'

4. What services should be added in the health centers to reach out to clients of sex workers?

Response: Health centers might not be the most appropriate setting to deliver specific services to men who pay for sex. Instead, other settings (e.g., bars and sex work venues) might be more amenable to interventions. Health centers should continue, however, to offer general HIV prevention services and attempt to better engage men in the HIV prevention and treatment cascades.

As a public health expert, this study is equality interesting to me as an evidence paper and as a study to guide policy recommendations. Unfortunately, the latter is missing.

Response: We thank the reviewer for highlighting these issues and encouraging us to improve our discussion on policy recommendations.

The goal of our study was to improve our understanding of the role of men who pay for sex in the complex HIV transmission dynamics arising from sex work.

We have been more specific with our policy recommendations by expanding our discussion (see previous responses above). We believe that the reviewer's suggested changes have strengthened the paper. Recognizing men who pay for sex as a priority population for HIV prevention is the first crucial step that will help national managers of HIV control programs assess the unmet prevention needs of these men.

Reviewer #3:
The study is very useful especially now that HIV prevalence remain stable in some countries in Sub Saharan Africa (SSA). It is appreciated to see that the authors have focused on a specific population group (Men) which provides more specific information regarding their vulnerability to HIV infections through pay for sex.

1. It is important to clarify starting with the abstract on which countries in SSA Africa the data was analyzed

> **Response:** We included data from 35 countries, and listing them all in the abstract is probably unwarranted. Instead, we compromised by stating that these 35 countries represent 95% of the male population 15-54 years of sub-Saharan Africa. Our addition on page 2 of the abstract is as follows:
>
> *'We included 87 surveys, totaling more than 368,000 male respondents (15-54 years old), from 35 countries that represent 95% of men in sub-Saharan Africa.'*
>
> A full list of countries included in the study is provided in Tables S1 – S2 on pages 2-10 of the supplement.

2. It is not clear on whether men who were involved in the study were heterosexual or not, I think the sexual orientation need to be stipulated clearly

> **Response:** We agree with the reviewer that the lack of specificity regarding sexual orientation is a limitation of our analyses. Unfortunately, the population-based surveys we used do not ask respondents about their sexual orientation and only 9 surveys did specify the female gender of the person respondents paid to have sex with. However, we believe that the great majority of respondents were heterosexual reporting on paid sex with women, since UNAIDS estimates that less than 2% of men in this region engage in same-sex behaviors. We have edited our discussion on page 19 to include this limitation:
>
> *'Third, most surveys do not specify the paid partner's gender when asking about paid sex and we cannot be certain that all men in our analytical sample have paid for sex with a woman. However, the proportion of men who have sex with men in this region is estimated to be less than 2% [50].'*
>
> We have also clarified in the Supplementary Materials on page 1 that only nine surveys specified paid sex with a woman, while the others did not specify partner sex.

3. It is also important that the authors clarify in the abstract section about whether men paid for sex with women or with men? this is not clearly stated in the abstract

Response: Only 9 surveys specified that the question referred to paid sex with a woman. The remaining ones did not, but we believe that only a minority of men would have engaged in paid sex with another man. Although we believe that the small impact of this limitation does not warrant a statement in the abstract, we now discuss these nuances in detail in on page 19 of our Discussion. However, we have edited the abstract to refer more generally to "*sex workers*" instead of "*female sex workers*".

4. An implication of the study on access to treatment will be useful

Response: Since our initial submission, we have been able to expand our analyses of antiretroviral use and viral load suppression by adding 2 DHS and 3 PHIA recent surveys, 2 of which collected this data. Despite this, the number of available surveys that collect this information remains low (N=9). In the updated analysis, we found that men who pay for sex living with HIV have similar levels of antiretroviral use and viral load suppression as compared to men who do not pay for sex, despite having higher HIV prevalence.

Given the small number of available surveys, we did not feel that it was appropriate to draw strong recommendations on access to treatment at this time.

5. Some additional lines that would contribute to the existing paradox that women also pay for sex (Mtenga et al. AIDS Res Ther (2018) 15:12 https://doi.org/10.1186/s12981-018-0199-6), will make a an important argument.

Response: Although we acknowledge that women can pay for sex, the gendered power and economic dynamics entail that the great majority of commercial sex acts are paid by men (Nancy Phaswana-Mafuya et al., 2014; Kuate-Defo 2004). In this context, we do not feel it is warranted to discuss this potentially marginal phenomenon.

References:

Phaswana-Mafuya N, Shisana O, Davids A, Tabane C, Mbelle M, Matseke G, et al. Perceptions of sugar mommy practices in South Africa. Journal of Psychology in Africa 2014; 24: 257-263.

Kuate-Defo B. Young people's relationships with sugar daddies and sugar mummies: what do we know and what do we need to know? African Journal of Public Health 2004; 8: 13-37.

Reviewer #4:
I would like to appreciate the authors for the great work. Conceptually, I found the paper very coherent and structured in a well thought out fashion.

1. First, motivation of the authors to do the paper has been mentioned as "less attention" drawn to interventions aimed at targeting clients of sex workers. However, this justification could not tell the convincing reason for doing the study as the study focuses on HIV epidemiology, including other related issues, among these groups. If you find that interventions on men paying for sex are given less attention, then the study should have been on finding reason for "why this happens". But now, your study is on HIV burden, HIV testing, condom use etc, and the driver for carrying out this should have been explained differently like for instance, limited knowledge base on this area.

> **Response:** We thank the reviewer for the opportunity to clarify the motivation for doing our study. As they have cited from paragraph 3 of our introduction, interventions prioritizing men who pay for sex are much less common than those for female sex workers. However, our motivation for the study extends beyond the consideration of “interventions”. Currently, UNAIDS does not recognize men who pay for sex as a priority population. This is partly because men who pay for sex do not face the stigma and other structural barriers of other key populations that exacerbate HIV acquisition and transmission risks. Nevertheless, lumping these men with the “partners of female sex workers” category does not enable programs to monitor and implement appropriate HIV interventions. By reviewing the HIV epidemiological data among men who pay for sex, we fill evidence gaps on several key indicators, including the population size, sexual behaviors, and burden of HIV, along with uptake of HIV prevention and treatment modalities, contributing to a better knowledge of the epidemic and hopefully better interventions. Having robust, descriptive measures of these indicators is essential to draw further international attention to this group and, ultimately, to develop interventions addressing the specific risks for this group.
>
> To address the reviewer’s point, we have edited our introduction on page 5 to explicitly mention the need to review comparable population-based epidemiological data on this population. This section now reads as follow:
>
> *‘Despite their central position in sexual networks, there has been comparatively less attention devoted to systematically reviewing representative epidemiological data on men who pay for sex and on interventions focused on this population.’*

Simply put, linking a study focused on HIV burden with a justification using a statement like inattention to prevention [of HIV among those groups] did not work here. Interventions might not be given attention but as the same time HIV burden can be known. So, this leads us to that, there might be many other important factors that potentially prevent policy makers and

intervention designers from drawing attention to interventions, and lack of knowledge on HIV epidemiology [the other issues this study has addressed] may still NOT be one of the factors.

Response: We agree with the reviewer that the lack of information on the HIV burden among men who pay for sex might not be the only factor explaining the lack of attention; other factors unrelated to their HIV burden may play a role. That said (as mentioned in our introduction), having a granular understanding of population sizes, sexual behaviors, HIV epidemiology, and uptake of HIV prevention interventions among this group is a necessary first step to accurately characterize HIV epidemics to inform policy makers and decide next best steps or questions to address. These data are also key inputs used by mathematical modelers to understand how best to optimize HIV prevention programs.

We have clarified in our introduction on page 5 that the lack of a systematic synthesis of standardized population-based surveys on HIV epidemiology among men who pay for sex was part of the motivation for conducting our study. We hope that these changes address the reviewer's concerns with the motivation of our study.

So, I suggest that it would be highly useful to succinctly explicate the direct contribution of the paper towards reducing men running into the paid sex practice as well as to our knowledge base on this area.

Response: We have revised our Discussion to highlight the direct contributions of our study. Specifically, we added on page 20 that:

'Strengths of this study include our exhaustive analysis of all available population-based surveys with information on men who pay for sex in SSA, without restriction to any survey type. We synthesized new information on the epidemiology of HIV and the HIV prevention and treatment cascades among men who paid for sex. Our large sample size allowed investigation of trends by regions and over time, and we estimated adjusted prevalence ratios using standardization to control for the effects of age and area of residence.'

It should also be noted that we do not argue that interventions should attempt to reduce the practice of paid sex among men. Throughout our discussion, we highlight the potential contribution of our paper to harm reduction interventions, to reduce the risk of HIV acquisition and transmission during sex work. Targeting structural determinants associated with sex work, such as the imbalance of power between female sex workers and their male clients, the criminalization of sex work, and social norms around the acceptability and use of condoms during paid sex, can help challenge social norms and structures that encourage sex work, and make the practice safer for sex workers by reducing the risk of stigmatization, violence, and HIV acquisition (Atuhaire 2021).

References:

Atuhaire L, Adetokunboh O, Shumba C, Nyasulu PS. Effect of community-based interventions targeting female sex workers along the HIV care cascade in sub-Saharan Africa: a systematic review and meta-analysis. Systematic Reviews. 2021 Dec;10(1):1-20.

2. Second, the selection process of the papers lacks some clarity. How did the authors restrict surveys between 2010-2020, for instance. It is also good to use PRIMSA adopted to the IPD, Individual Participant Data meta-analysis.

Response: We have now completed and provided a copy of the *Meta-analyses Of Observational Studies in Epidemiology* (MOOSE) checklist, which contains items that are more appropriate for assessing our specific methodology than the PRISMA IPD checklist.

With regards to the selection process, in this analysis we did not use traditional systematic review methods to search for surveys. This is because the population-based surveys we were interested in are not indexed in bibliographic databases.

Instead, we extensively searched the DHS, AIS, PHIA, MICS and other survey sources (KAIS, SABSSM, BAIS, EPHS, NAIIS, etc.) for all population-based surveys conducted in sub-Saharan African countries between 2000-2020. We identified 227 surveys, of which 87 contained information on paid sex ever. The 2000-2020 period was chosen because few surveys were available from before 2000 and only a single DHS survey (Zimbabwe 1999) asked questions about paid sex before the year 2000. We are therefore confident that we have included almost all available surveys which included data on paid sex ever among men.

3. Third, the chosen of the random effect model was not justified; why not fixed effect model rather. Give reason.

Response: A random effects model assumes that the observed survey estimates (e.g., population size) vary across surveys because of real differences in the estimate of the outcome in each survey setting (i.e., each survey can have their own "true" estimate of the outcome). In contrast, a fixed effects model assumes that all surveys are estimating the same estimate of the outcome, thus that the underlying surveys all have the same "true" estimate. Given the high heterogeneity observed in some of our outcomes, a random effect model is more appropriate.

4. I found the I-squared statistics to be extremely high, suggesting considerable between-study variation in terms of the variables you studied. Given this variability, do you think producing a

single overall estimate through pooling is a good practice, as the papers are too different already.

Response: Indeed, the I^2 statistic represent the proportion of the variation in the observed survey estimates that is due to variation in the "true" survey estimates. An implicit assumption of random effects models is that variation in observed estimates is due to variation in the true estimates; therefore, I^2 statistics tend to be higher when using random effects models. High I^2 is also often used as justification for using a random effects model.

We agree that heterogeneity is very high. However, we do not provide one single overall estimate for our estimands. Rather, we provide pooled estimates for each of the four SSA regions and further investigated if time could explain part of this heterogeneity through meta-regression. It is unlikely that the survey instruments are "too different" for them to be pooled since questions are largely standardized. Further, we have used standardization by age and urban/rural residence type to improve our comparisons of ratios which would have reduced heterogeneity in the underlying estimates. We provided pooled estimates despite the high heterogeneity because we believe that there is indeed a large underlying variation in some of these estimates.

5. Meta-analysists often try fitting meta-regression to explain the variation; in this study, meta-regression could have been done better by including more confounding variables in the model to get unbiased results. Also, under variables, make it clear whether all of these are outcome variables, and mention your independent variables, if any.

Response: We used meta-regressions and stratified analyses to examine the role of region and time in explaining the observed heterogeneity. Our goal was not to draw causal relationships between selected exposure and outcomes. Rather, it was to see if the estimates of prevalence or association differed by selected variables. Although we did not report it in the paper, we have attempted to examine the impact of socio-economic status on the estimated proportions and prevalence ratios. Unfortunately, the sample size within each wealth quintile in each country was not sufficient to draw robust inferences.

6. Fourth, of the three design elements of a complex design study like DHS, the authors take into account only clustering and unequal probability of selection, via, weighting. However, stratification is missing, and failing to take this problem into account would result in estimation of standard errors that are biased. DHS experts highly recommend researchers to make account of all the three design elements of complex designs like DHS. I would see as a big limitation of the paper as your confidence intervals are likely to be biased.

Response: We thank the author for pointing out this omission. We have now included stratification in our analyses. The Methods section was edited on page 7 to clarify this as follows:

'Using respondent-level data from each survey, we calculated relevant estimands accounting for the complex survey design (i.e., survey weights, stratification, and clustering).'

7. Finally, these days, countries are moving towards ensuring health equality within their population. International agreements like SDG calls for equity; that means, while the time is to look at health outcomes between different population groups within a country, your study was about aggregating country-level information into a regional level. How do you see the implication of the paper for the wellbeing of different segments of population in each individual country in light of SDG?

Response: The reviewer is correct to point out the importance of the SDG "leave no one behind" slogan. While we would have liked to examine within-country variation in population size and HIV prevalence among men who paid for sex, there were too few estimates per country to pool at the country-level. For this same reason, we were not able to further stratify by sub-national areas or socio-economic status. However, it should be noted that, by restricting our study sample to men who ever paid for sex, we are already drawing attention to a hidden population, providing new information on a group of men that is usually "left behind".

We have also expanded our analysis to look at the prevalence of paying for sex ever and in the past 12 months by age groups and residence types; these results can be found in Tables S5 and S6. We have also added the following to page 10 of our Results section:

'Men residing in urban areas were more likely to report ever paying for sex (9·7%, 95%CI: 7·3-12·7) than those from rural areas (7·1, 95%CI 5·2-9·6; Table S6) . . . Younger men (15-24 years) were more likely to report paying for sex in the past 12 months (5·1%, 95%CI: 3·6-7·1%) than those aged 35-54 years (2·2%, 95%CI: 1·5-3·2%; Table S7).'

Within country inequality analysis of HIV prevalence among men paying for sex is very important to understand who these group of men are and why they are being engaged in the activity. Because, all men in this high-risk behavior are not homogenous population group and how their engagement in this risky sexual behavior is affected by contexts they live in remains a huge research question that could substantially contribute towards the SDG related with HIV. To the contrary, you did aggregate based analysis. Can explain more on this.

Response: We thank the reviewer for this question. Despite our large sample size of over 368,000 men, the sample size of men who ever paid for sex by sub-national areas

becomes too small to enable robust analyses. For example, roughly 9% of participants reported ever paying for sex and HIV prevalence among this group was close to 6%. In other words, the average sample size of sexually active men per survey was close to 4,200 (368,000/87 surveys) and only 380 men per survey (4,200*9%) reported paying for sex, of which roughly 20 (380*6%) would be living with HIV. With this small sample size, conducting sub-national analyses is not possible. It is because of these considerations that we conducted aggregated analyses.

Finally, investigating individual contexts and reasons for paying for sex requires different evidence base than the one provided in the surveys considered in this study. Importantly, qualitative research in which men who pay for sex are asked to explain why and how they engage in paid sex may be better suited to answering this research question.

Requests from the Editors:
1. In the title, please move the dates before the colon.

Response: The title has been edited to read: '*HIV prevalence, population sizes, and HIV prevention among men who paid for sex in sub-Saharan Africa (2000-2020): a meta-analysis of 87 population-based surveys.'*

2. Please combine the "Methods" and "Findings" subsections of your abstract.

Response: These subsections of the abstract have been combined.

3. Please add a new final sentence to the combined subsection, which should begin "Study limitations include ..." or similar and should quote 2-3 of the study's main limitations.

Response: We have added the following sentence to the abstract regarding study limitations:

'Study limitations include reliance on self-reports of sensitive behaviors and the small number of surveys with information on ART and VLS."

4. Please remove the information on funding from the abstract.

Response: This information has been removed.

5. After the abstract, we will need to ask you to add a new and accessible "Author summary" section in non-identical prose. You may find it helpful to consult one or two recent research papers published in PLOS Medicine to get a sense of the preferred style.

Response: This section has been added to the manuscript.

6. Early in the Methods section, please state whether the analysis was registered and whether or not it had a protocol or prespecified analysis plan, and if so attach the document(s) as a supplementary file(s), referred to in the text.

Response: As we did not use traditional systematic review methodology in this analysis, our protocol was not registered. We have added a sentence in the Methods section to clarify this: *'The analysis was not registered.'*

7. Throughout the text, please style reference call-outs as follows: "... may explain this [30,31]." (noting the absence of spaces within the square brackets).

Response: The in-text references have been edited to match this style.

8. Please remove the information on funding and competing interests from the end of the main text. In the event of publication, this will appear in the article metadata, via entries in the submission form.

Response: This information has been removed.

9. In the reference list, please convert italics and boldface to plain text.

Response: The reference list has been re-formatted to plain text.

10. Noting reference 8 and others, please list 6 author names rather than 3, followed where appropriate with "et al.".

Response: The reference list has been updated to include 6 author names where applicable.

11. Noting reference 32, please add "[preprint]" to all preprints cited.

Response: This article has been published since our original submission and the reference has been updated to reflect this.

12. Please use the journal name abbreviation "PLoS ONE" in the reference list.

Response: This abbreviation has been corrected in the reference list.

13. Please complete a checklist for the most appropriate reporting guideline, e.g., PRISMA (we suggest PRISMA 2020, https://journals.plos.org/plosmedicine/article?id=10.1371/journal.pmed.1003583), and attach this as a supplementary file, labelled "S1_PRISMA_Checklist" or similar and referred to as such in your Methods section.
In the checklist, please refer to individual items by section (e.g., "Methods") and paragraph number, not by line or page numbers as these generally change in the event of publication.

Response: We have completed the *Meta-analyses Of Observational Studies in Epidemiology* checklist (MOOSE) and have attached this in a supplementary file labelled "S1_MOOSE_Checklist". We have referred to this in the Methods section as follows: '*This meta-analysis was reported in accordance with MOOSE guidelines [27].'*
